# Learning prevalent patterns of co-morbidities in multichronic patients using population-based healthcare data

**DOI:** 10.1038/s41598-024-51249-7

**Published:** 2024-01-25

**Authors:** Chiara Seghieri, Costanza Tortù, Domenico Tricò, Simone Leonetti

**Affiliations:** 1 Management and Healthcare Laboratory, Institute of Management and Department EMbeDS, Sant’Anna School of Advanced Studies, Piazza Martiri della Libertà 33, 56127 Pisa, Italy; 2 Department of Clinical and Experimental Medicine, University of Pisa, Via Roma 67, 56126 Pisa, Italy; 3 Management and Healthcare Laboratory, Interdisciplinary Research Center “Health Science”, Sant’Anna School of Advanced Studies, Piazza Martiri della Libertà 33, 56127 Pisa, Italy

**Keywords:** Health policy, Public health

## Abstract

The prevalence of longstanding chronic diseases has increased worldwide, along with the average age of the population. As a result, an increasing number of people is affected by two or more chronic conditions simultaneously, and healthcare systems are facing the challenge of treating multimorbid patients effectively. Current therapeutic strategies are suited to manage each chronic condition separately, without considering the whole clinical condition of the patient. This approach may lead to suboptimal clinical outcomes and system inefficiencies (e.g. redundant diagnostic tests and inadequate drug prescriptions). We develop a novel methodology based on the joint implementation of data reduction and clustering algorithms to identify patterns of chronic diseases that are likely to co-occur in multichronic patients. We analyse data from a large adult population of multichronic patients living in Tuscany (Italy) in 2019 which was stratified by sex and age classes. Results demonstrate that (i) cardio-metabolic, endocrine, and neuro-degenerative diseases represent a stable pattern of multimorbidity, and (ii) disease prevalence and clustering vary across ages and between women and men. Identifying the most common multichronic profiles can help tailor medical protocols to patients’ needs and reduce costs. Furthermore, analysing temporal patterns of disease can refine risk predictions for evolutive chronic conditions.

## Introduction

Health systems around the world are facing significant challenges due to ageing populations as well as long-term chronic diseases which are recognised as the leading causes of death and disability worldwide^1^. According to the World Health Organization (WHO), 74% of deaths worldwide are due to non-communicable diseases (NCDs): among these, cancers, respiratory diseases and diabetes account for over 80% of all premature deaths, occurring in patients between 30 and 69 years^2^. NCDs also negatively impact patients’ overall quality of life^3^, as individuals affected by a chronic disease are required to set restrictions on their daily actions, adjust their habits, follow personalised clinical and pharmaceutical protocols, and be monitored through frequent medical visits. Moreover, chronic diseases heavily impact national health systems, given the expensive and longstanding clinical support delivered to patients^4^.

Chronic conditions are likely to co-occur, particularly in older patients. *Multimorbidity*, here taking the definition of the co-existence of two or more chronic diseases in the same patient^5,6^, is estimated to affect 60% of people aged 65 or above in Europe^7^, imposing a significant burden on patients, healthcare systems and society in general^8–11^. Furthermore, treating a multichronic patient poses significant challenges for healthcare professionals due to the complex clinical needs of the patients. For example, treatments or drugs may be effective for a given chronic disease but they may also cause negative side effects that worsen the clinical condition related to another co-existing chronic disease. Most healthcare systems globally lack medical protocols regarding the management of multichronic patients. Indeed, healthcare systems are still organised based on the Single Disease Model (SDM)^12^ which can result in lower quality healthcare support to multichronic patients as well as reduced overall system efficiency^13^. From an individual and a public perspective, it would be beneficial to treat multichronic patients with a personalised holistic approach that extends beyond the standard SDM. It would clearly be unfeasible for healthcare professionals to develop specific medical protocols for all the possible combinations of chronic conditions that can affect a multichronic patient. However, a viable strategy would be to identify the most frequent patterns of chronic diseases that are likely to co-occur in multichronic patients and prioritise them for the development of specific clinical protocols.

Various methods have been proposed for characterizing multimorbidity. While conventional strategies rely on a priori knowledge such as cause-and-effect relationships and common risk factors^14^, the value of data-driven approaches becomes apparent. Rooted in clustering algorithms, these strategies^15,16^ group diseases or patients based on data similarities, offering a more dynamic and potentially insightful perspective compared to traditional approaches. Recent studies pursuing the data-driven approach have employed either clustering algorithms (both hard clustering algorithms^17–26^—such as hierarchical clustering and K-means—and soft clustering algorithms^27–32^, allowing entities to belong to multiple clusters) or data mining tools^18,19,27,28,33–35^ such as principal component analysis (PCA)^36–38^, multi-correspondence analysis (MCA)^39,40^, exploratory factor analysis (EFA)^41,42^, latent class analysis (LCA)^43–45^, or the visual-oriented t-distributed stochastic neighbor embedding (t-SNE)^46–49^ algorithm to explore relationships among chronic conditions, providing insights into critical factors, data variability, and distinct group classifications. The current body of research investigating patterns of chronic conditions has notable limitations. First, there is a lack of consensus on the optimal analytical methods for identifying disease patterns in a multichronic population, as different approaches^15^ are employed across the literature. Second, many studies^50,51^ rely on data extracted from admission hospital records or self-reported data, the latter being unsupervised by clinical assessments and susceptible to measurement errors. Additionally, a common trend in existing studies is the concentration on older populations, typically excluding patients younger than 60, further restricting the generalizability of findings.

In this investigation, we aimed to address the data-related limitations of previous research by leveraging comprehensive administrative health data spanning a broad age range (encompassing individuals aged 25 to 100 years) and to address the methodology-related limitations by integrating data mining tools and clustering algorithms. In particular, this study integrates visually oriented data mining tools and soft clustering algorithms to detect prevalent patterns of chronic conditions within a sizable multimorbid population, using population-based healthcare data in the Tuscany region, Italy, in 2019. The methodological approach is structured into two main phases: first, it involves a case-finding algorithm based on administrative regional data^52,53^ to detect chronic conditions by adopting a purely data driven approach; then, it integrates a data reduction algorithm—the t-SNE algorithm^46–49,54^—and a clustering algorithm—fuzzy k-means (FkM)^55,56^—to identify patterns of chronic diseases that are likely to co-occur in multichronic patients. The primary input of the proposed integrated methodology was a co-occurrence-based distance matrix, in which each element measures the Jaccard’s difference^57^ between pairs of diseases, obtained by considering the number of multichronic patients who are jointly affected by the two conditions (co-occurrences). The ultimate objective is to provide healthcare professionals and systems with interpretable evidence, enhancing comprehension of disease patterns and aiding in the identification of recommended resource prioritisation and clinical attention. This paper is organised as follows: “Data and methods” section describes data and methods, focusing on the main methodological aspects which includes the data sources, the steps of the clustering, and the sub-clustering analysis; “Results” section presents the main results; “Discussion” section discusses the main findings and outlines policy implications; and “Conclusions” section concludes and outlines future perspectives.

## Data and methods

### The Tuscan healthcare system

In this study, the analysis focuses on all residents enrolled in the healthcare system of Tuscany region (Italy). Italy has a regionally-based national health service which provides universal coverage largely free of charge at the point of delivery^58^. Tuscany is a large region in central Italy that is home to approximately 3.7 million people (6.2% of the Italian population)^59^. The Tuscan healthcare system is a non-competitive system in which patients are free to choose any provider and which consists of 3 local health authorities, 4 university hospitals, and 26 health districts. In Tuscany, approximately 90% of healthcare services are administered and delivered by public providers and/or private accredited providers.

### Data sources

In this study, we conducted a population-based analysis using individual-level administrative data from the Tuscany region for 2019. The Tuscan healthcare administrative databases include data regarding all public and private accredited healthcare providers. Specifically, this study used six data sources: (i) hospital inpatient data; (ii) emergency care data; (iii) outpatient care data; (iv) drug prescription data; (v) exemption data; and (vi) socio-demographic data on all residents enrolled in the Tuscan healthcare system, including sex, date of birth, and date of death^60^. The data are pseudo-anonymised at the regional health information system office where each patient is assigned a unique identifier applying to all administrative databases. This identifier does not include the patient’s identity or other sensitive data.

The study approval by the conjoint ethics committee of Scuola Superiore Sant’Anna di Pisa and Scuola Normale di Pisa (Italy) was also obtained (Delibera no. 20/2020). The study was carried out in compliance and accordance with the General Data Protection Regulation (2016/679) and the Italian Legislative Decree No. 196/2003 (“Personal Data Protection Code”) within the project CoNtAcT—Accountable networks for chronic populations: new governance mechanisms to promote professional integration and value improvement—a 3-year project funded by Bando Ricerca Salute 2018 of Tuscany Region (Italy) for supporting the regional bodies in the performance evaluation and organization of the healthcare system. The informed consent for using data is not necessary according to the Italian Decree No. 196/2003 (“Personal Data Protection Code”), art. 2 sexies.

### Identification of the population of multimorbid patients

Specific case-finding algorithms^52^ were applied to the data discussed above to identify prevalent multichronic populations. This procedure enabled us to identify the population of multimorbid patients to be included in the analysis. The population of interest included individuals who, in 2019, were affected by two or more chronic diseases and were between the ages of 25 and 100 years old. Sub-populations of patients were identified by stratifying the sample according to age group (25–44, 45–64, 65–84, and 85–100 years) and sex (male (M) and female (F)). In compliance with the standardised definition of rare diseases for European countries^61^, chronic conditions with a prevalence lower than 0.05% in each population were not considered.

### Co-occurrences-based distance among diseases

Let us denote *k* as the generic chronic condition included in the analysis, where k takes values from 1 to the maximum number of diseases considered in the analysis; the set *K* collects the chronic conditions. The population *N* represents the population of multimorbid patients between 25 and 100 years of age, where each individual is indexed with *i*, and *i* can take values from 1 to the sample size of each population. Let *C*_*ik*_ be the binary variable describing whether the patient *i* is affected by the chronic disease k: hence, *C*_*ik*_ = *1* if patient *i* is affected by disease *k*, *C*_*ik*_ = 0 otherwise. The *K* dimensional vector ***C***_***i***_ collects the *C*_*ik*_ elements and fully characterises the chronic condition of individual *i*. We also define the (*N* × *K)* dimensional matrix ***C*** obtained by binding the individual specific vectors ***C***_*i*_ by row. The matrix ***C*** is employed to obtain a (*K* × *K)* dimensional distance matrix ***D***, where each cell *d*_*kl*_ measures the likelihood of chronic condition k to co-occur with disease l. We perform Jaccard’s distance^57^ on the transpose of the ***C*** matrix to obtain a (*K* × *N*) matrix, where diseases and patients are represented in rows and columns, respectively. The values reported in each cell of ***D*** range from 0 to 1. Values closer to 0 provide strong evidence that the two diseases co-occur in the population of multimorbid patients for which we account; in contrast, values that are closer to 1 suggest that the co-occurrence of the two diseases is weak.

### Clustering and sub-clustering analysis of multimorbidity

Given the distance matrix ***D***, we use clustering to group chronic diseases that are more likely to co-occur, according to the data. The goal is to identify clusters of diseases such that diseases within the same cluster are more likely to co-occur than diseases that are referred to different clusters. The classification of diseases follows a purely data-driven approach, without accounting for any a priori knowledge about chronic conditions or multimorbidity. In this study, we use fuzzy clustering (a soft clustering approach)^62–64^ to detect fuzzy groups of diseases that are likely to co-occur. Specifically, we use the FkM algorithm^65^ to classify diseases into fuzzy clusters, while estimating the degrees of membership of each disease to the clusters (membership degrees range from 0 to 1). Note that in the FkM algorithm, the number of clusters must be specified a priori by the researcher: to select the best number of clusters to be employed by the FkM algorithm, we preliminarily implement a hard clustering algorithm on the data and we select the optimal number of clusters according to the results of the Elbow rule^66^ and Silhouette analysis^67^. The fuzzy approach enables us to interpret a probability measure of chronic diseases to be assigned to each of the clusters and to differentiate between those that are classified in a given group with a strong membership degree, and those that are not well classified (because their probability of being assigned to the clusters is not significantly high). Following the same approach employed in many studies on data mining and clustering algorithms^68–73^, we implement a data reduction algorithm before performing the clustering analysis. Specifically, we first apply the t-SNE algorithm^74^ on the distance matrix ***D***, to map diseases on a bi-dimensional domain, and then we implement the FkM on the coordinates of the points obtained by the algorithm. t-SNE is based on the stochastic neighbour embedding (SNE) algorithm^75^ and is very effective with high dimensional data because it reduces the dimensionality of the analysis but still explains the whole variability in the data. Once we obtain the t-SNE coordinates ***z***_***k***_ = (*x*_*k*_*, y*_*k*_), we compute the optimal number of clusters according to the Elbow criterion and the Silhouette analysis and we apply the FkM algorithm to obtain fuzzy clusters of chronic diseases. The FkM algorithm is an iterative machine learning algorithm that minimizes an objective function, which is based on (i) a fuzzy parameter *m*, and (ii) the sum of the squared distances between each disease *i*, characterised by the t-SNE coordinates, and the centroids of the clusters. If we denote *G* as the total number of clusters, with the generic cluster indexed as *g*, with *g* = (1,…,*G*), the objective function (OF) to be minimised by the algorithm is expressed by

$${\text{OF}}\left({\text{FkM}}\right)=\sum \limits_{{\text{k}}:1}^{{\text{K}}}\sum \limits_{{\text{g}}:1}^{{\text{G}}}{\left({{\text{u}}}_{{\text{kg}}}\right)}^{{\text{m}}}\times {{\text{d}}}_{{\text{kg}}}^{2},$$

where *u*_*kg*_ is the degree of membership of disease *k* in cluster *g*, *d*^2^_*kg*_ is the squared distance between disease *k* and the centroid of cluster *g*, and *m* is the fuzzy parameter (here we set *m* to the standard value, 2). The function is minimised under the constraint that the degrees of membership of each individual are required to sum up to 1, such that ${\sum }_{g:1}^{G}{u}_{kg}=1$ for each disease *k*. The numerical solution of the problem is computed using the expectation–maximization (EM) algorithm.

The steps of the FkM algorithm can be summarized as follows:

1. Fix the fuzzy parameter *m* and the number of clusters *G*, generate an initial random partition ***U***^**(0)**^ and compute the corresponding centroids.
  In each iteration *r.*
2. Compute the centroids of the *G* groups using the following equation:
  $${C}_{g}^{\left(r\right)}=\frac{{\sum }_{k:1}^{K}{\left({u}_{kg}\right)}^{m{z}_{k}}}{{\sum }_{k:1}^{K}{\left({u}_{kg}\right)}^{m}}.$$
3. If there exist some *g* such that *d*_*kg*_ = 0, then set *u*_*kg*_ = 1 and *u*_*k j*_ = 0 for all the groups *j* such that *j *$\ne$* g*, otherwise use the following equation:
  $${u}_{kg}^{\left(r\right)}=\frac{1}{{\sum }_{j:1}^{G}{\left[\frac{{d}_{kg}}{{d}_{kj}}\right]}^{\frac{2}{m-1}}}.$$
4. Repeat steps 2 and 3 until the difference ***U***^**(*r*)**^** − *U***^**(*r***−**1)**^ is z lower than an arbitrary constant.

When the algorithm reaches convergence, it returns a (*K* × *G*) matrix ***U*** where the generic element *u*_*kg*_ reports the membership degree of disease *k* to the cluster *g.* The disease *k* is considered to be well classified into the cluster *g* if the corresponding membership degree *u*_*kg*_ exceeds 0.5.

To further explore the co-occurrence of chronic diseases within multimorbid patients, we perform a secondary clustering analysis on each of the generated macro clusters (this approach is known as sub-clustering), by following the same procedure described above. The clustering and sub-clustering analysis is performed both on the whole population of multimorbid patients and on the sub-populations of patients generated by stratifying the sample according to age classes and sex.

### Software and packages

Data management of administrative data was conducted using SAS v.9.4 software. Data mining, cluster analysis and statistical analysis were performed using R v.4.1.2 software and packages^65,76–87^.

## Results

**Figure 1:**
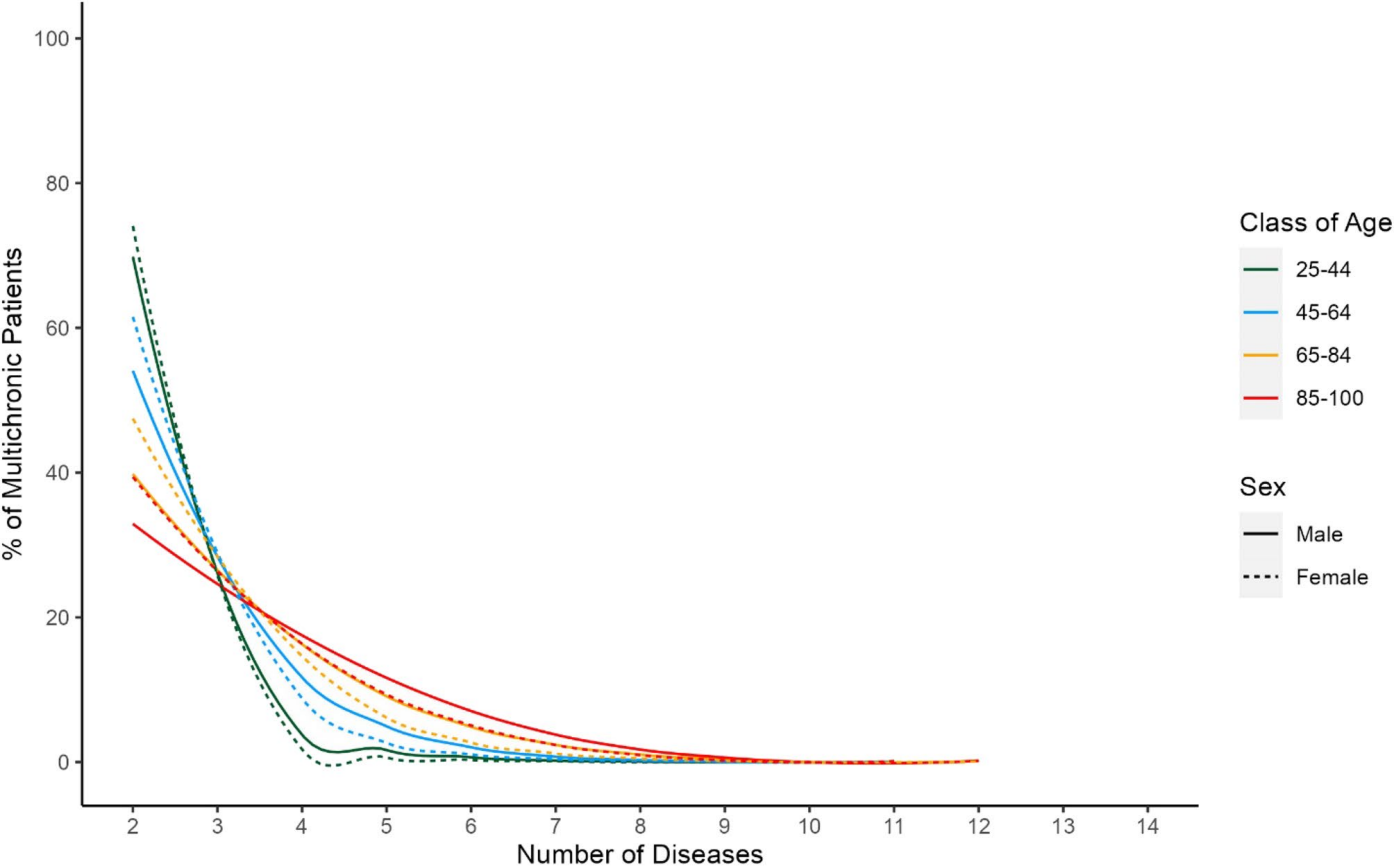
Number of co-occurring diseases in each sub-population.

The entire multimorbid population included 636,754 patients (53.98% women). The average age was 73.1 (± 13.4) years, and the most prevalent age group was 65–84 years. Figure 1 provides a graphical representation of the severity of multimorbidity across sub-populations identified by sex and age class. Most patients were affected by 3 or more co-occurrent diseases, the number of which increased with age. Moreover, the number of co-occurring diseases was higher in men than in women. Further details on the descriptive statistics are available in the Supplementary Appendix Tables S1 and S2.

The clustering analysis returned several macro clusters; the structure of these clusters was further explored through sub-clusters. The same methodology was performed both on the whole population of multimorbid patients and on the sub-populations stratified by sex and age class. In this section, we present and discuss results related to the analysis performed on the whole population, while only summarising the main findings of sub-analyses. Complete results related to all the analysed sub-populations are provided in the Supplementary Appendix Figs. S1–S8 and Supplementary Appendix Tables S4–S7). The tables summarising the results of the clustering and sub-clustering analyses provide information on the cluster prevalences and on the prevalences of the singular diseases included in each cluster. Cluster prevalences are computed as the ratio between the number of multimorbid patients included in that given cluster and the size of the whole multimorbid population. Note that each cluster is named according to the most frequent chronic conditions that it includes. In addition, on the graphs, diseases are labelled with abbreviations, which are fully described in the Supplementary Appendix Table S3.

**Figure 2:**
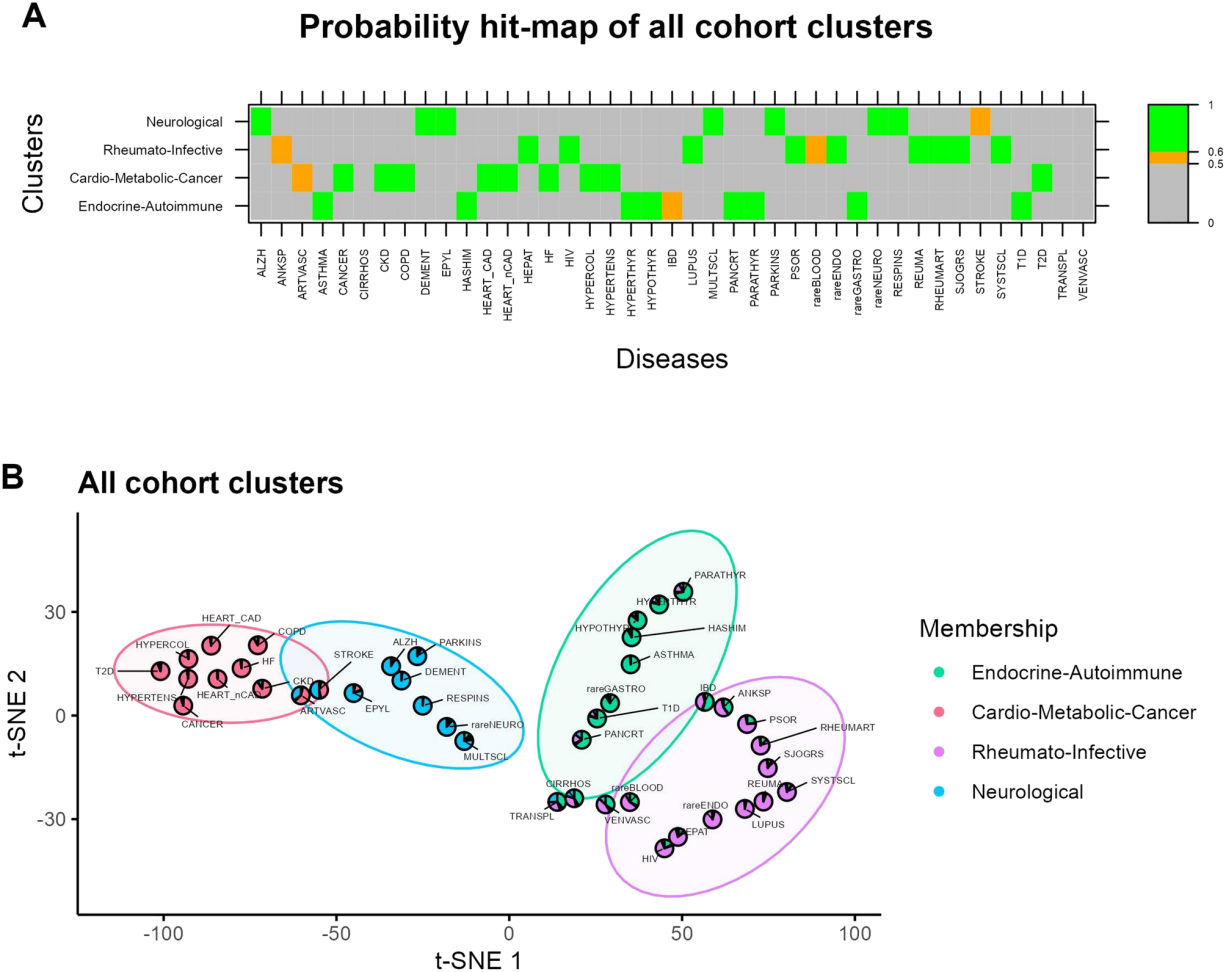
Clustering analysis on the whole population. Multimorbidity representation in the overall Tuscan population: (**A**) Hit-map of membership degree of each degree to the clusters. Colours identify thresholds of the membership degrees: grey (< 0.5), orange (0.5–0.6) and green (> 0.6). (**B**) Scatter pie plot of identified clusters in two dimensions using t-SNE and FkM. The pie related to each disease is coloured proportionally to the membership degrees of that disease to the clusters*.*

Figure 2 presents the results related to the main analysis performed on the whole population. Here, we identify four macro-clusters, including Cardio-Metabolic-Cancer diseases, Endocrine-Autoimmune diseases, Neurological diseases, and Rheumato-Infective diseases, ordered by prevalence. The most prevalent clusters in the observation population are the Cardio-Metabolic-Cancer cluster (76.85%) and the Endocrine-Autoimmune cluster (7.00%). The prevalence of Cardio-Metabolic diseases in the entire population and sub-populations was higher in men than in women, for both older and younger patients. Five diseases were excluded from the whole population analysis due to their low prevalence (Table 1). Heart diseases, metabolic diseases and cancer are included in the Cardio-Metabolic-Cancer cluster; asthma, type 1 diabetes, inflammatory bowel disease (IBD) and thyroid syndromes comprise the Endocrine-Autoimmune cluster; brain and neurodegenerative diseases define the neurological cluster; finally, rheumatoid arthritis, psoriasis, hepatitis and HIV are included in the Rheumato-Infective cluster.

**Table 1:** Sub-clustering analysis on the whole population.

Whole population				
Cluster attributes				
Cardio-metabolic-cancer	Endocrine-autoimmune	Neurological	Rheumato-infective	Not assigned
**(76.85%)**	**(7.00%)**	**(2.67%)**	**(0.7%)**	**N.A.**
**Sub-c1 (5.57%)**	**Sub-c1 (0.03%)**	**Sub-c1 (0.63%)**	**Sub-c1 (0.41%)**	CIRRHOS *(0.27%)*
ARTVASC *(1.23%)*	PANCRT *(0.05%)*	EPYL *(0.35%)*	ANKSP *(0.07%)*	TRANSPL *(0.12%)*
CKD *(1.25%)*	rareGASTRO *(0.15%)*	STROKE *(2.21%)*	LUPUS *(0.06%)*	VENVASC *(0.36%)*
COPD *(2.27%)*	T1D *(0.10%)*		PSOR *(0.29%)*	
HF *(2.53%)*		**Sub-c2 (0.64%)**	REUMA *(0.07%)*	
	**Sub-c2 (6.55%)**	ALZH *(0.44%)*	RHEUMART *(0.31%)*	
**Sub-c2 (70.07%)**	ASTHMA *(0.84%)*	DEMENT *(0.37%)*	SJOGRS *(0.13%)*	
CANCER *(5.67%)*	HASHIM *(1.34%)*	PARKINS *(0.48%)*	SYSTSCL *(0.05%)*	
HEART_CAD *(3.39%)*	HYPERTHYR *(0.59%)*			
HEART_nCAD *(5.84%)*	HYPOTHYR *(3.66%)*	**Sub-c3 (0.04%)**	**Sub-c2 (0.15%)**	
HYPERCOL *(7.62%)*	IBD *(0.35%)*	MULTSCL *(0.13%)*	HEPAT *(0.46%)*	
HYPERTENS *(16.63%)*	PARATHYR *(0.08%)*	rareNEURO *(0.08%)*	HIV *(0.10%)*	
T2D *(5.52%)*		RESPINS *(0.17%)*	rareBLOOD *(0.06%)*	
			rareENDO *(0.10%)*	
Prevalence < 0.05% (MYASTH, rareCONGEN, rareCVD, rareSKIN, TRANSPL2YRS)				

Numbers in brackets refer to the cluster and sub-cluster prevalences (bold) or to disease-specific prevalences (italics). Rare diseases (population prevalence lower than 0.05%) that are excluded from the analysis are indicated in the bottom row. Diseases whose membership degree resulting from the FkM algorithm is lower than 0.5 are reported as “Not Assigned”.

Most of the diseases were classified in one of the clusters, while a few of them could not be uniquely classified into one group only (panel A). By following the conventional approach used in interpreting results from fuzzy clustering, we considered a disease uniquely classified in one cluster if its corresponding membership degree to that cluster exceeded 0.5. Here, hepatic cirrhosis, transplants and venous vasculopathy did not present any membership degree that met this criterion. Hepatic cirrhosis and venous vasculopathy were fuzzy classified into the Endocrine-Autoimmune and the Rheumato-Infective clusters, while transplants were associated with these two groups and the Neurological cluster.

Table 1 presents the results related to the sub-clustering analysis of the whole population. The sub-clustering analysis identified two or three sub-clusters within each macro cluster defined in the first step of the analysis. The table also provides information on the sub cluster prevalences. The sub-cluster 2 in the Cardio-Metabolic-Cancer cluster represents the most prevalent sub-group of diseases observed. For the Endocrine-Autoimmune cluster, the most prevalent diseases were grouped into subcluster 2, which includes asthma, IBD and thyroid syndromes. The Neurological cluster was divided into 3 groups; sub-cluster 1 and sub-cluster 2 were the most prevalent and included epilepsy and stroke diseases, and neurodegenerative diseases, respectively. The Rheumato-Infective macro cluster was divided into two sub-clusters: sub-cluster 1 included rheumatological diseases, while sub-cluster 2 included hepato-infective diseases.

Table 2 summarises and compares the main findings obtained in four sub-populations of multichronic patients: women 25–44 years old, men 25–44 years old, women 65–84 years old and men 65–84 years old. In these sub-populations, chronic conditions were grouped according to four or five clusters, only very few of which were not uniquely classified. In younger patients, regardless of sex, the chronic diseases that were more likely to co-occur were hypertension, type 2 diabetes (T2D), hypercholesterolemia, and heart disease (both ischemic and non-ischemic)—all of which were included in the Cardio-Metabolic cluster. In older patients, these pathological conditions were also associated with heart failure (HF), stroke, chronic obstructive pulmonary disease (COPD), chronic kidney disease (CKD), and cancer. The Endocrine cluster included the most prevalent thyroid syndromes (hypothyroidism, prevalence 3.66%), asthma, IBD and type 1 diabetes (T1D). These pathological conditions were more prevalent in women than in men, particularly in younger patients, where they frequently co-occurred with cancer. In older patients, endocrine diseases were merged into a single cluster with infective diseases or were grouped in the Cardio-Metabolic cluster. Autoimmune diseases were all co-located in one cluster, but in older populations they were likely to co-occur with rheumatic conditions (men) or pulmonary diseases (women). The Neurological cluster was observed in all sub-populations and consisted of neurological and brain diseases. Neurodegenerative diseases were minimally represented in younger patients (prevalence < 0.05%), while Alzheimer, Parkinson, and dementia become increasingly prevalent after 65 years of age. The Rheumato-Infective clusters consisted of the less prevalent diseases in the entire population. Rheumatological diseases were mostly represented by rheumatoid arthritis and psoriasis and were more prevalent in older patients and in women. Oncological diseases are clustered differently across age classes. In younger patients, cancer was less prevalent and was included in the Endocrine cluster. At 65 years and older, however, cancer clustered with the Cardio-Metabolic group.

**Table 2:** Clustering analysis showing sub-populations, including descriptions of the identified macro-clusters in four meaningful sub-populations of multimorbid patients.

Class of age	Sex										
	Male					Female					
	Cluster attributes					Cluster attributes					
	Cardio-metabolic (24.13%)	Cancer-endocrine (16.79%)	Autoimmune (5.46%)	Neurological (2.34%)	Not assigned	Cancer-endocrine (22.30%)	Cardio-metabolic (6.55%)	Autoimmune (2.06%)		Neurological (2.02%)	Not assigned
25–44	HEART_CAD	CANCER	ASTHMA	ARTVASC	CKD	CANCER	CKD	ASTHMA		ARTVASC	HASHIM
	HEART_nCAD	HASHIM	HEPAT	EPYL		HYPERTHYR	HEART_nCAD	rareGASTRO		EPYL	HEPAT
	HF	HYPOTHYR	HYPERTHYR	STROKE		HYPOTHYR	HYPERCOL	T1D		rareENDO	LUPUS
	HYPERCOL	PSOR	IBD			IBD	HYPERTENS			STROKE	REUMA
	HYPERTENS	TRANSPL	rareGASTRO			MULTSCL	T2D			VENVASC	
	T2D		T1D			PSOR					
						RHEUMART					

	Cluster attributes					Cluster attributes					
	Cardio-metabolic-cancer (91.74%)	Endocrine-infective (1.35%)	Autoimmune-rheumato (0.66%)	Neurological (0.62%)	Not assigned	Cardio-metabolic-cancer-endocrine (89.27%)	Neurological (3.97%)	Rheumato (0.72%)	Bowel-autoimmune (0.03%)	Pulmonary-autoimmune (0.03%)	Not assigned
65–84	ARTVASC	HASHIM	ANKSP	ALZH	MULTSCL	CANCER	ALZH	CIRRHOS	ANKSP	MYASTH	ASTHMA
	CANCER	HEPAT	ASTHMA	DEMENT	rareNEURO	COPD	ARTVASC	LUPUS	IBD	PANCRT	HEPAT
	CKD	HIV	CIRRHOS	EPYL	RESPINS	HASHIM	CKD	MULTSCL	rareSKIN	rareNEURO	PARATHYR
	COPD	HYPERTHYR	IBD	PARKINS	T1D	HEART_CAD	DEMENT	PSOR		RESPINS	rareBLOOD
	HEART_CAD	HYPOTHYR	PANCRT			HEART_nCAD	EPYL	rareCVD		T1D	rareGASTRO
	HEART_nCAD	MYASTH	PSOR			HF	PARKINS	rareENDO			
	HF	PARATHYR	rareBLOOD			HYPERCOL	STROKE	REUMA			
	HYPERCOL	rareENDO	rareCVD			HYPERTENS	VENVASC	RHEUMART			
	HYPERTENS	rareGASTRO	RHEUMART			HYPERTHYR		SJOGRS			
	STROKE		TRANSPL			HYPOTHYR		SYSTSCL			
	T2D		VENVASC			T2D		TRANSPL			

In sub-population analyses, several diseases could not be assigned to a unique cluster. For example, chronic hepatitis was partially associated with Neurological and Autoimmune clusters in women, but in older patients, it was likely to co-occur with diseases belonging to two additional clusters (Rheumato and Cardio-Metabolic-Cancer-Endocrine clusters). In older women, hyper- and hypo-parathyroidism were mostly associated with the Cardio-Metabolic-Cancer-Endocrine cluster, although its membership degree was lower than the 50% threshold. Moreover, asthma was associated with both the Cardio-Metabolic-Cancer-Endocrine and Bowel-Autoimmune clusters. In the sub-population of men aged 25–44, most diseases were uniquely identified in specific clusters, except for chronic kidney disease, which showed a fuzzy association with the Cardio-Metabolic and the Neurological clusters. Finally, Multiple sclerosis, rare neurologic diseases, respiratory insufficiency and T1D were associated with both the Autoimmune-Rheumato and Endocrine-Infective clusters in older men.

## Discussion

In this study, we developed a data-driven and population-based approach using the joint implementation of a data reduction algorithm and a fuzzy cluster algorithm to identify the most common patterns of multimorbidity in the Tuscan multimorbid population and in different sub-populations of patients. We observed a slightly higher multimorbidity rate among females than among males; however, stratifying by the cumulative number of diseases, this ratio changes in the older age groups, in which the coexistence of four or more chronic diseases is more prevalent among males. These trends are in agreement with those described in the current literature^88–90^.

Our findings reveal three relevant and recurrent patterns of multimorbidity in all the populations analysed. First, Cardio-metabolic diseases represent a stable pattern of multimorbidity that can affect even relatively young patients. In older patients, cardio-metabolic diseases are also associated with other conditions such as heart failure, cancer, kidney disease, and COPD, which may share the same risk factors or represent the natural consequences of the pre-existing diseases in youths. The second cluster includes thyroid syndromes; these pathologies are likely to be associated with cancer in young patients, but they are grouped with Cardiometabolic or rheumatoid diseases in older patients. The third emerging cluster groups brain and neuro-degenerative diseases, which are differently associated across age classes: stroke and epilepsy jointly are prevalent in young and middle-age patients, while Alzheimer, Parkinson, and dementia are likely to co-occur in older-age patients.

Cluster analyses stratified by sex and age were mostly consistent with the analysis of the whole population, confirming similar disease clusters, but still presenting some notable differences. The first element that differs is the group of the rare diseases that are excluded from the analysis due to their low prevalence. Some diseases are very rare in the first decades of life and are consequently removed from the subgroup analysis of young multimorbid patients. In particular, coronary heart diseases are extremely rare in young women, while rheumatoid and neurodegenerative diseases show negligible prevalence in both sexes. Lupus is a rare condition in men of all ages, while in women it becomes significantly prevalent after 45 years of age. The second element that differs in the sub-populations is related to the cluster size, given that clusters in the younger sub-populations are generally smaller in terms of number of diseases included in the groups than those obtained while analysing older patients. Above age 65, the higher number of diseases included in each cluster could indicate a temporal sequence of events. For example, HF appearing in the Cardio-Metabolic cluster of elderly patients, regardless of sex, suggests a cause-effect relationship with other conditions that are already present in the same cluster at younger ages, such as hypertension and T2D; this finding aligns with current pathophysiology knowledge^91,92^. The third element that differs in sub-clustering results involves the composition of the clusters themselves. Cancer is more strongly associated with endocrine diseases in young patients and with cardiometabolic and pulmonary diseases in older patients; this may be explained by the increasing prevalence of colorectal and lung cancer across all ages. These cancers are associated with unhealthy lifestyles and tobacco smoking, which in turn are linked to metabolic diseases and COPD^93–95^. The autoimmune cluster has no defined disease pattern across all ages; however, it is still possible to identify different sub-patterns of co-occurring diseases such as IBD and T1D, or asthma and T1D in younger classes of ages, or IBD and rheumatoid diseases in age classes over 65 years. Neurodegenerative diseases such as dementia, Alzheimer and Parkinson are more prevalent in elderly patients. Dysfunction in memory circuits due to the ageing of neurons may explain the association of Alzheimer and Parkinson diseases with dementia^96,97^. Epilepsy is one of the less prevalent diseases and its association with stroke is stable in young and middle-aged sub-clusters; this association could be explained by the post-stroke epilepsy phenomenon^98,99^.

It is difficult to compare the results obtained in this study with other findings available in the existing literature, since the ensemble of diseases which is included in the analyses varies, as well as the data sources and employed methods^15^. Most of the available studies performed their analyses on populations characterised by much smaller sample sizes (usually less than 200.000 individuals) often restricted to older patients and with prevalences based on self-reported questionnaires. The present study differentiates from these existing studies in two main elements. First, we analysed data from a larger population including young, adult, and elderly patients between 25 and 100 years of age. Second, we used a case-finding algorithm to detect chronic conditions applied to administrative health records^52^.

Despite the differences in sampling identification and methodologies, some similarities emerged in our findings compared with previous studies using data-driven approaches to detect patterns of co-occurring chronic diseases. For example, the results obtained in two systematic reviews, Prados-Torres et al.^50^ and Busija et al.^51^, implemented a patterns analysis of multimorbidity can be somehow compared with our findings. In particular, Prados-Torres et al.^50^ identified 97 multimorbidity profiles reported in 14 separate studies and highlighted three groups of partially comparable patterns: the first group combines cardiovascular and metabolic diseases, the second group is characterised by diseases related to mental health disorders, and the third group includes musculoskeletal diseases. Busija et al.^51^ identified 407 multimorbidity profiles over 51 studies that match the inclusion criteria and results of 98 distinct analyses of multimorbidity profiles. The analysis produced five interpretable patterns, including a group that embraces respiratory disease (chronic obstructive pulmonary disease and asthma), a group that covers skeletal-muscle-soft tissue disorders (osteoporosis, back pain, musculoskeletal disorders, and soft tissue disorders), and a group that incorporates neurodegenerative disorders (Parkinson’s disease and dementia). Thus, these studies both identified cardio-metabolic, musculoskeletal, and neurodegenerative disorders as relevant patterns of multimorbidity. Similarly, in the present study, we detected the Cardio-Metabolic and Neurological clusters in both the whole population of multimorbid patients and in most of the sub-population analyses. Moreover, the cluster related to musculoskeletal disorders resembles the cluster of rheumatoid diseases, herein identified in association with endocrine syndromes, particularly in older ages. Poblador-Plou et al.^100^ conducted a comparative study of multimorbid patterns between two European patient populations. The sample covered 79,291 Dutch patients and 275,682 Spanish patients between 15 and 65 years of age whose data were extracted from electronic medical records and analysed using EFA. The study results are partly consistent with our findings, which were obtained following a more robust method on a much larger sample, although they are not completely superimposable. In fact, Poblador-Plou et al.^100^ detected clusters related to rheumatologic and bowel diseases in all sub-populations of patients stratified by age and sex, and noted that respiratory, neuro-degenerative, and hematopoietic diseases become more prevalent in the elderly. They also observed that cardio-metabolic diseases were likely to affect both younger and older men, while women were likely to be affected in older ages only. An opposite relationship emerged from the analysis of thyroid diseases; they were more frequent in women of all ages and in older men.

### Limitations and strengths

The present study has some limitations. Although administrative healthcare data have been increasingly used in the health services and policy research over the years^101^, these data may be susceptible to data incompleteness and a lack of useful clinical information (i.e. date of diagnosis and gravity of the disease). In particular, the lack of the date of diagnosis prevents the determination of a temporal collocation to the onset of any given chronic condition in multichronic patients, hindering the opportunity of examining the chronological order in which diseases occur in the same patient. Additionally, administrative data do not provide any information about laboratory biomarkers for a deeper understanding of the mechanisms of morbidities. However, this study also offers several important contributions. First, it covers a large population, since it includes the whole population of multimorbid patients enrolled in the Tuscan healthcare system. Second, this work follows a purely data-driven approach. The joint usage of a data mining tool for data visualization—the t-SNE algorithm—and of a fuzzy clustering algorithm—the FkM algorithm, which directly operates on the coordinates returned by t-SNE—allowed us to obtain results that are both rigorous and easily interpretable. Moreover, the fuzzy perspective enabled us to explicitly consider the uncertainty in the classification and differentiate diseases that are uniquely classified in one cluster from those that are not well classified.

### Policy implications

Given the limitations inherent in current therapeutic approaches, which are often centred on a monolithic view of chronic conditions, lacking a comprehensive consideration of the patient’s overall clinical profile, our research proposes potential avenues to encourage a more holistic understanding of multimorbidity. To illustrate, healthcare providers can devise innovative strategies founded on:

(i) *Waste reduction* by identifying prevalent multichronic profiles, healthcare systems can streamline diagnostic procedures, minimizing redundancy and eliminating unnecessary tests. This approach contributes to efficient resource use and allocation among populations.
(ii) *Refined risk predictions* detecting common combinations of chronic conditions can empower healthcare professionals to proactively address potential complications, leading to enhanced risk management and more effective prevention strategies.
(iii) *Patient-centered care* healthcare providers can tailor interventions and programs based on a patient’s specific multichronic profiles and their needs. Indeed, professionals should be trained to work together in interdisciplinary teams to design and deliver comprehensive treatment strategies to align care pathways with the needs and expectations of the identified clusters of patients. On the other hand, patients with an elevated risk of experiencing multiple chronic conditions can enhance their awareness regarding proactive measures to prevent and manage the onset of related chronic diseases. This may involve educational interventions and programs targeting individuals with specific profiles focused on self-management and on preserving health through, for instance, health lifestyle improvements such as engaging in regular physical activity, adopting a personalized diet, and steering clear of any potential risk factors that could contribute to the development of additional chronic conditions.
(iv) *Enhanced clinical efficacy* integrating knowledge about prevalent multichronic profiles into clinical decision-making processes can enhance the formulation of informed and effective strategies that are shared by the multiprofessional teams. This, in turn, holds the potential to avoid harm and improve outcomes for patients with multimorbidity.

However, the future implications of our research extend beyond individualized patient care, offering potential benefits for the global healthcare system. For instance, the waste reduction and efficient resource utilization, driven by a targeted and comprehensive approach to multimorbidity, hold the potential to alleviate the pressure on healthcare budgets on a larger scale and could result in substantial cost savings for healthcare systems worldwide. Similarly, the integration of knowledge about prevalent multichronic profiles into clinical decision-making processes may lead to a reduction in hospitalizations and the mitigation of complications. This shift towards a more proactive and targeted approach to multimorbidity could contribute to the overall resilience and sustainability of healthcare systems worldwide. In conclusion, embracing a more holistic understanding of multimorbidity not only benefits individual patients but holds the potential to transform and optimize the functioning of global healthcare systems, with the final aim of fostering a change to Value-Based Health Care.

## Conclusions

Effective and efficient management of multimorbidity is one of the greatest challenges facing patients, health systems, and society in general. This is particularly true for healthcare systems attempting to shift to the provision of high-value care for their population. Indeed, multimorbidity poses a high treatment burden and a significant impairment on patients’ quality of life. Growing complexity in appointment schedules, diagnostic exams, and treatment plans—which are often conflicting—can result in incomplete adherence to therapeutic interventions and suboptimal outcomes, which could be improved by coordinating clinical decisions among different specialists. Our study proposes an original approach to analyse the relationships between chronic diseases in a large and well-characterised population to support data-informed decision-making in the development of guidelines and effective care delivery models for multimorbidity management. Additionally, these results should increase awareness among health professionals about the burden of multimorbidity in chronic patients, which increases with age. The high prevalence of some disease combinations should prompt active screening of associated medical conditions when a first disease is diagnosed, and close longitudinal surveillance and preventive actions should be implemented for early recognition and to delay the development of these conditions.

Future research related to the current topic is needed to better understand the burden of multimorbidity on hard outcomes such as death, disability, patterns of service utilisation and costs (both direct and indirect). Further insight will help support targeted interventions in the organisation of care delivery, the effective and population-based allocation of resources and in the promotion of quality care.

### Supplementary Information

Supplementary Information.

## Data Availability

Third-party data were used to generate the results reported in this paper, specifically the authors used pseudo-anonymised administrative data of Tuscany region (Italy) which are not publicly available. The administrative data are analysed by the authors under an agreement between the regional administration—Direzione Diritti di Cittadinanza e Coesione Sociale—of Tuscany (Italy) and Sant’Anna School of Advanced Studies for supporting the regional bodies in the performance evaluation of the healthcare system. The agreement does not involve any ethical board. The data that is accessible to interested researchers is aggregate and a dataset in aggregate format is available upon request to corresponding author (simone.leonetti@santannapisa.it) to replicate the study results.
