# Supplementary material for: Learning prevalent patterns of co-morbidities in multichronic patients using population-based healthcare data
Source: Sci Rep. 2024 Jan 25;14:2186. doi: 10.1038/s41598-024-51249-7 (PMC10810806; doi:10.1038/s41598-024-51249-7)
Supplement: Supplementary file 1 — Supplementary Information. [file 41598_2024_51249_MOESM1_ESM.docx]

**Online Appendix**

1. **Descriptive statistics**

Appendix Table S1: Descriptive table of male, female, and overall populations.

| **Characteristics** | **Male** | **Female** | **All** |
| --- | --- | --- | --- |
|  | N=293,061 (46.02%) | N=343,693 (53.98%) | N=636,754 (100%) |
| **Age (Years)**  Mean (SD) | 72.7 (*±*12.5) | 73.5 (*±*14.1) | 73.1 (*±*13.4) |
| Median [p25% - p75%] | 74 [65 - 82] | 75 [65 - 84] | 75 [65 - 83] |
| **Class of Age (N (%))**  25-44 | 6,833 (2.33 %) | 12,421 (3.61 %) | 19,254 (3.02 %) |
| 45-64 | 64,326 (21.95 %) | 71,926 (20.93 %) | 136,252 (21.40 %) |
| 65-84 | 171,612 (58.56 %) | 178,892 (52.05 %) | 350,504 (55.05 %) |
| 85-100 | 50,093 (17.09 %) | 79,603 (23.16 %) | 129,696 (20.37 %) |
| **Number of Diseases (N (%))** | | | |
| 2 | 126,676 (43.23 %) | 174,678 (50.82 %) | 301,354 (47.33 %) |
| 3 | 73,605 (25.12 %) | 87,617 (25.49 %) | 161,222 (25.32 %) |
| 4 | 43,763 (14.93 %) | 42,653 (12.41 %) | 86,416 (13.57 %) |
| 5 | 24,804 (8.46 %) | 21,319 (6.20 %) | 46,123 (7.24 %) |
| 6+ | 24,213 (8.26 %) | 17,426 (5.07 %) | 41,639 (6.54 %) |

Appendix Table S2: Descriptive table for class of ages in male and female populations.

|  | **Male** | | | |  | **Female** | | | |
| --- | --- | --- | --- | --- | --- | --- | --- | --- | --- |
| **Characteristics** | **25-44** | **45-64** | **65-84** | **85-100** |  | **25-44** | **45-64** | **65-84** | **85-100** |
|  | N=6,833 (2.33%) | N=64,326 (21.95%) | N=171,612 (58.56%) | N=50,093 (17.09%) |  | N=12,421 (3.61%) | N=71,926 (20.93%) | N=178,892 (52.05%) | N=79,603 (23.16%) |
| **Age (Years)** |  |  |  |  |  |  |  |  |  |
| Mean (SD) | 37.4 (±5.6) | 57.3 (±5.1) | 75.0 (±5.5) | 89.0 (±3.4) |  | 37.3 (±5.4) | 56.7 (±5.4) | 75.4 (±5.6) | 89.8 (±3.7) |
| Median [p25% - p75%] | 39 [33 - 42] | 58 [54 - 62] | 75 [71 - 80] | 88 [86 - 91] |  | 38 [33 - 42] | 57 [53 - 61] | 76 [71 - 80] | 89 [87 - 92] |
| **Number of Diseases (N (%))** |  |  |  |  |  |  |  |  |  |
| 2 | 4,961 (72.60 %) | 36,018 (55.99 %) | 69,164 (40.30 %) | 16,466 (32.87 %) |  | 10,000 (77.29 %) | 46,369 (64.47 %) | 86,914 (48.58 %) | 31,423 (39.47 %) |
| 3 | 1,267 (18.54 %) | 15,661 (24.35 %) | 44,255 (25.79 %) | 12,381 (24.72 %) |  | 2,118 (17.05 %) | 16,365 (22.75 %) | 47,907 (26.78 %) | 21,016 (26.40 %) |
| 4 | 390 (5.71 %) | 7,249 (11.27 %) | 27,283 (15.90 %) | 8,800 (17.57 %) |  | 487 (3.92 %) | 5,745 (7.99 %) | 23,616 (13.20 %) | 12,638 (15.88 %) |
| 5 | 143 (2.09 %) | 3,316 (5.15 %) | 15,471 (9.02 %) | 5,847 (11.67 %) |  | 147 (1.18 %) | 2,148 (2.99 %) | 11,395 (6.37 %) | 7,570 (9.51 %) |
| 6+ | 72 (1.05 %) | 2,082 (3.24 %) | 15,439 (9.00 %) | 6,599 (13.17 %) |  | 69 (0.56 %) | 1,299 (1.81 %) | 9,060 (5.06 %) | 6,956 (8.74 %) |

Appendix Table S3: Descriptive table of diseases abbreviations.

| Tag | Description |
| --- | --- |
| ALZH | ALZHEIMER |
| ANKSP | ANKYLOSING SPONDYLITIS |
| ARTVASC | ARTERIAL VASCULOPATHY |
| ASTHMA | ASTHMA |
|  |  |
| CANCER | CANCER |
| CIRRHOS | HEPATIC CIRRHOSIS |
| CKD | CHRONIC KIDNEY DISEASE-RENAL REPLACEMENT THERAPY |
| COPD | CHRONIC OBSTRUCTIVE PULMONARY DISEASE |
|  |  |
| DEMENT | DEMENTIA |
|  |  |
| EPYL | EPILEPSY |
|  |  |
| HASHIM | HASHIMOTO’S THYROIDITIS |
| HEART_CAD | CORONARY ARTERY DISEASE |
| HEART_nCAD | non-ischemic HEART DISEASE |
| HEPAT | CHRONIC HEPATITIS |
| HF | HEART FAILURE |
| HIV | HIV POSITIVE AND AIDS CONCLUDED |
| HYPERCOL | HYPERCHOLESTEROL |
| HYPERTENS | HYPERTENSION |
| HYPERTHYR | HYPERTHYROIDISMS |
| HYPOTHYR | HYPOTHYROIDISM |
|  |  |
| IBD | INFIAMMATORY BOWEL DISEASE |
|  |  |
| LUPUS | SYSTEMIC LUPUS ERYTHEMATOSUS |
|  |  |
| MULTSCL | MULTIPLE SCLEROSIS |
| MYASTH | SEVERE MYASTHENIA |
|  |  |
| PANCRT | CHRONIC PANCREATITIS |
| PARATHYR | HYPER AND HYPOPARATHYROIDISM |
| PARKINS | PARKINSON’S DISEASE |
| PSOR | PSORIASIS AND PSORIASIC ARTHROPATHY |
|  |  |
| rareBLOOD | BLOOD AND HEMATOPOIETIC ORGANS DISEASES |
| rareCONGEN | CONGENITAL MALFORMATIONS |
| rareCVD | CIRCULATORY SYSTEM DISEASES |
| rareENDO | ENDOCRINE GLANDS, NUTRITION, METABOLISM AND IMMUNE DISORDERS |
| rareGASTRO | DIGESTIVE SYSTEM DISEASES |
| rareNEURO | NERVOUS SYSTEM AND SENSE ORGANS DISEASES |
| rareSKIN | SKIN AND SUBCUTANEOUS TISSUE DISEASES |
| RESPINS | RESPIRATORY INSUFFICIENCY / OXYGEN THERAPY |
| REUMA | OSTEOMUSCULAR SYSTEM AND CONNECTIVE TISSUE DISEASES |
| RHEUMART | RHEUMATOID ARTHRITIS |
|  |  |
| SJOGRS | SJOGREN SYNDROME |
| STROKE | STROKE |
| SYSTSCL | SYSTEMIC SCLEROSIS |
|  |  |
| T1D | TYPE 1 DIABETES |
| T2D | TYPE 2 DIABETES |
| TRANSPL | TRANSPLANTS (10 years) |
| TRANSPL2YRS | RECENT TRANSPLANTS (2 years) |
|  |  |
| VENVASC | VENOUS VASCULOPATHY |

1. **Clustering and sub-clustering analysis in the sub-populations identified by common sex and age class.**
   1. **Clustering analysis**

Appendix Figure S1: Clustering analysis, females 25-44. Multimorbidity representation in the population including females aged between 25 and 44 years: A) Hit-map of membership degree of each degree to the clusters. Colors identify thresholds of the membership degrees gray (<0,5), orange (0,5-0,6) and green (>0,6); B) Scatter pie plot of identified clusters in two dimensions using t-SNE and FkM. The pie related to each disease is colored proportionally to the membership degrees of that disease to the clusters.


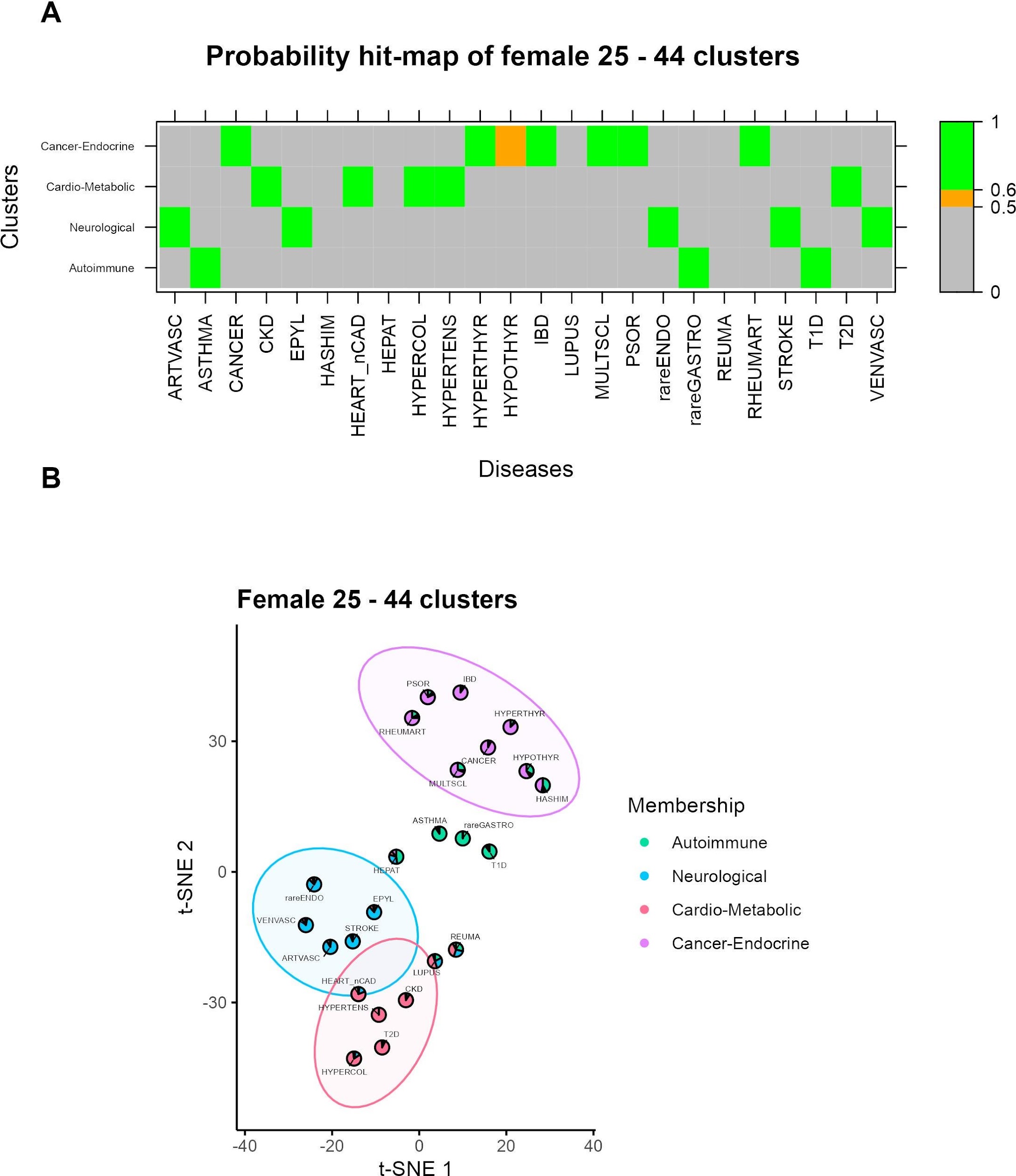


Appendix Figure S2: Clustering analysis, females 45-64. Multimorbidity representation in the population including females aged between 45 and 64 years: A) Hit-map of membership degree of each degree to the clusters. Colors identify thresholds of the membership degrees gray (<0,5), orange (0,5-0,6) and green (>0,6); B) Scatter pie plot of identified clusters in two dimensions using t-SNE and FkM. The pie related to each disease is colored proportionally to the membership degrees of that disease to the clusters.


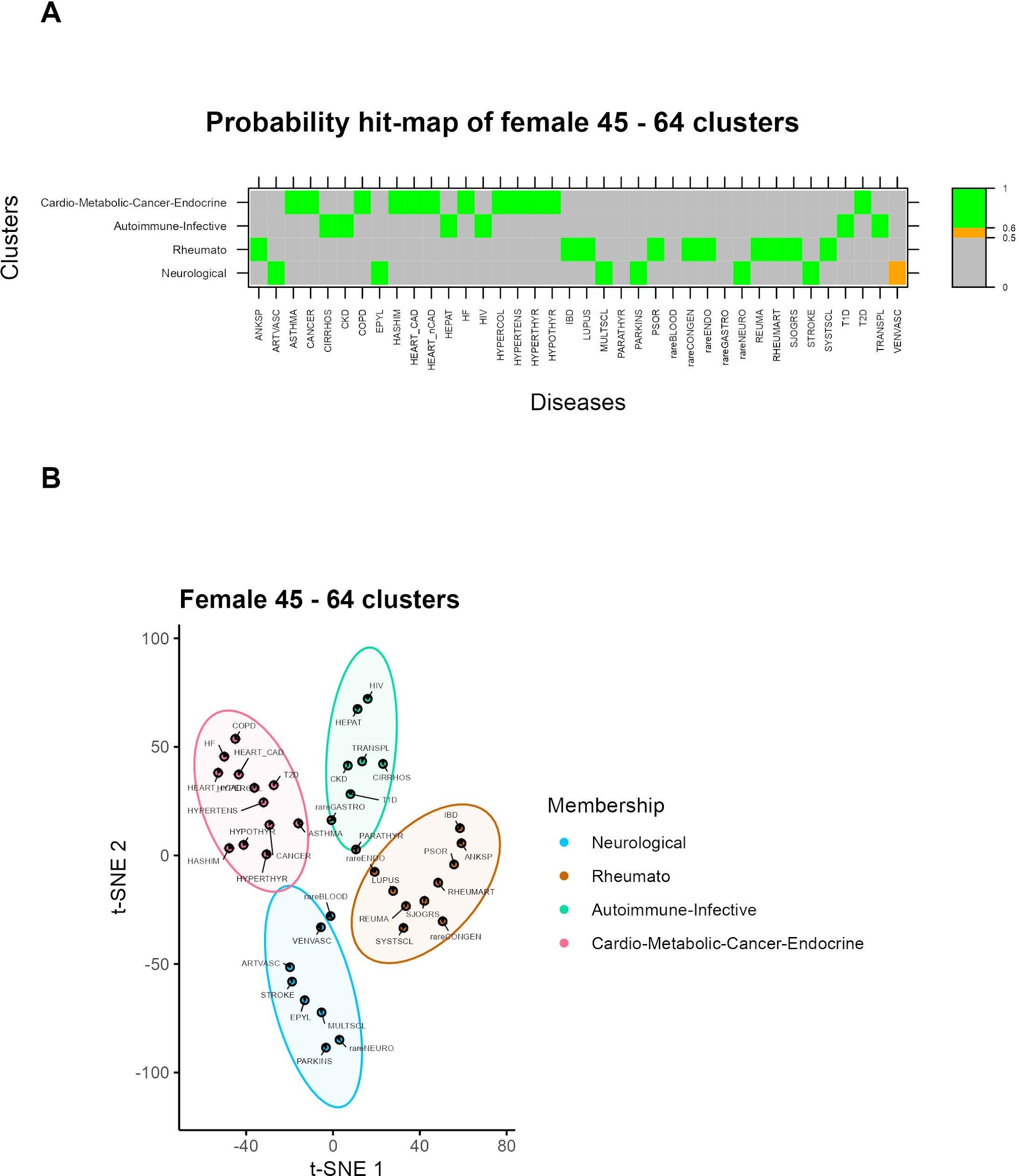


Appendix Figure S3: Clustering analysis, females 65-84. Multimorbidity representation in the population including females aged between 65 and 84 years: A) Hit-map of membership degree of each degree to the clusters. Colors identify thresholds of the membership degrees gray (<0,5), orange (0,5-0,6) and green (>0,6); B) Scatter pie plot of identified clusters in two dimensions using t-SNE and FkM. The pie related to each disease is colored proportionally to the membership degrees of that disease to the clusters.


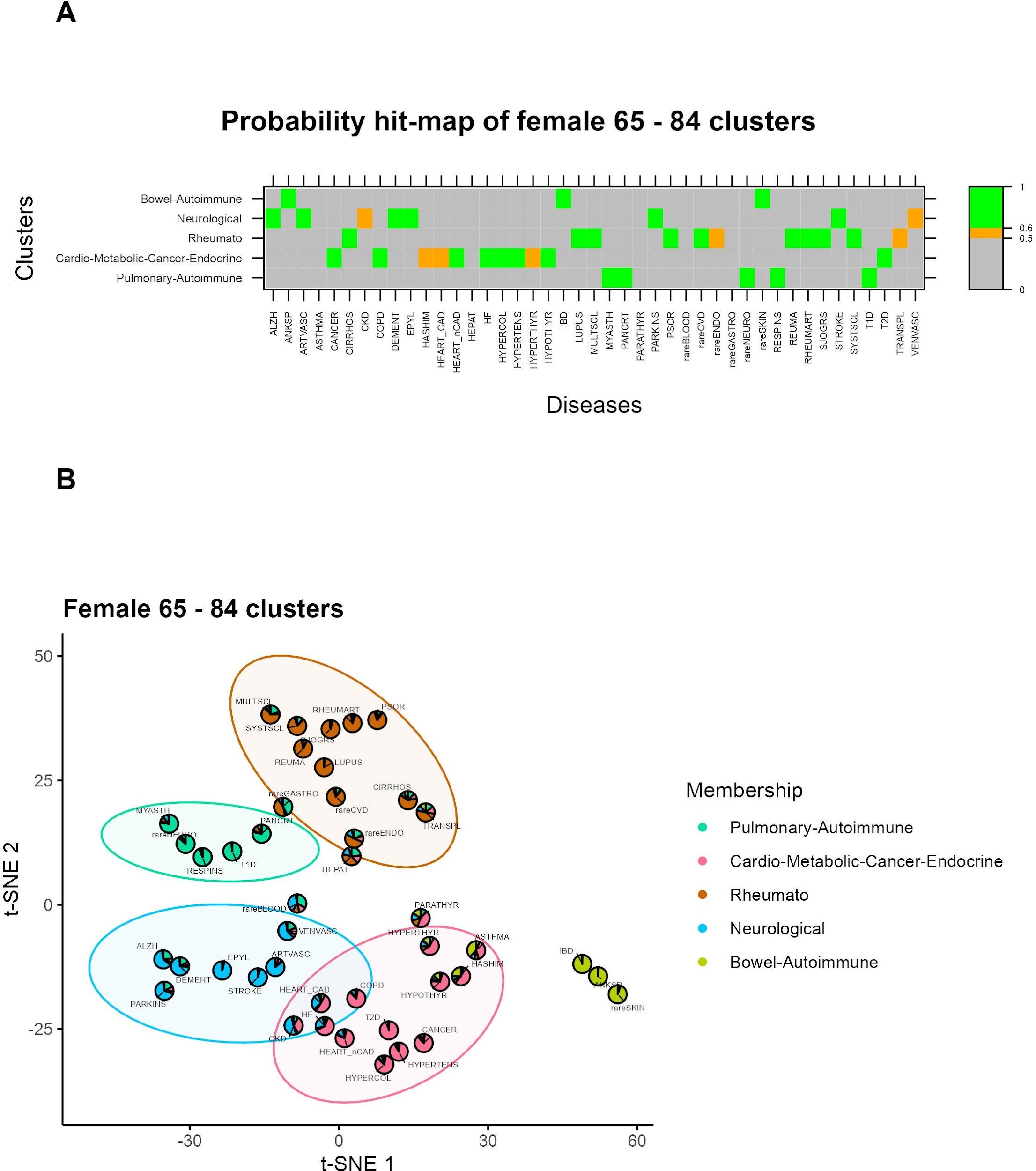


Appendix Figure S4: Clustering analysis, females 85-100. Multimorbidity representation in the population including females aged between 85 and 100 years: A) Hit-map of membership degree of each degree to the clus- ters. Colors identify thresholds of the membership degrees gray (<0,5), orange (0,5-0,6) and green (>0,6); B) Scatter pie plot of identified clusters in two dimensions using t-SNE and FkM. The pie related to each disease is colored proportionally to the membership degrees of that disease to the clusters.


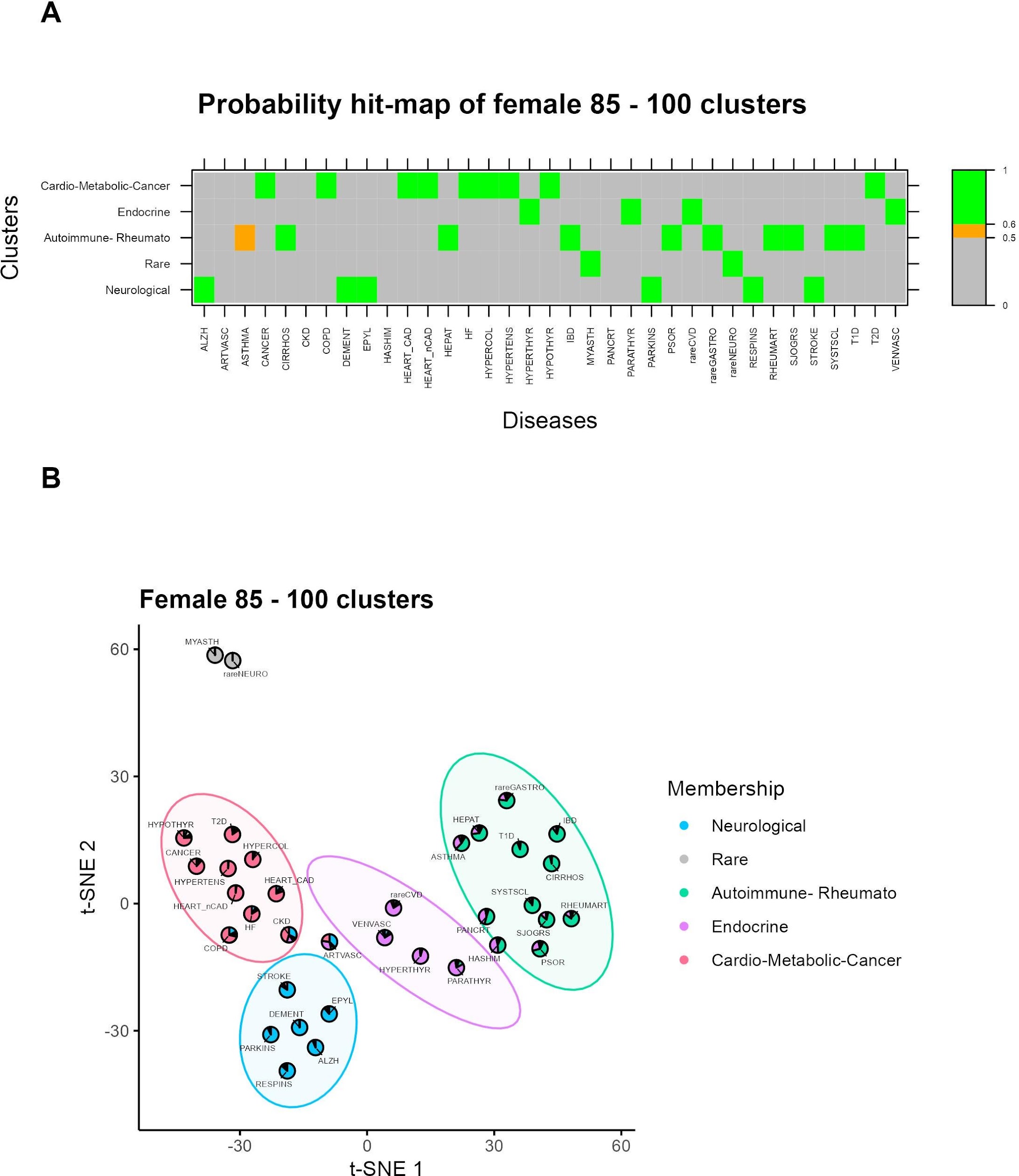


Appendix Figure S5: Clustering analysis, males 25-44. Multimorbidity representation in the population including males aged between 25 and 44 years: A) Hit-map of membership degree of each degree to the clusters. Colors identify thresholds of the membership degrees gray (<0,5), orange (0,5-0,6) and green (>0,6); B) Scatter pie plot of identified clusters in two dimensions using t-SNE and FkM. The pie related to each disease is colored proportionally to the membership degrees of that disease to the clusters.


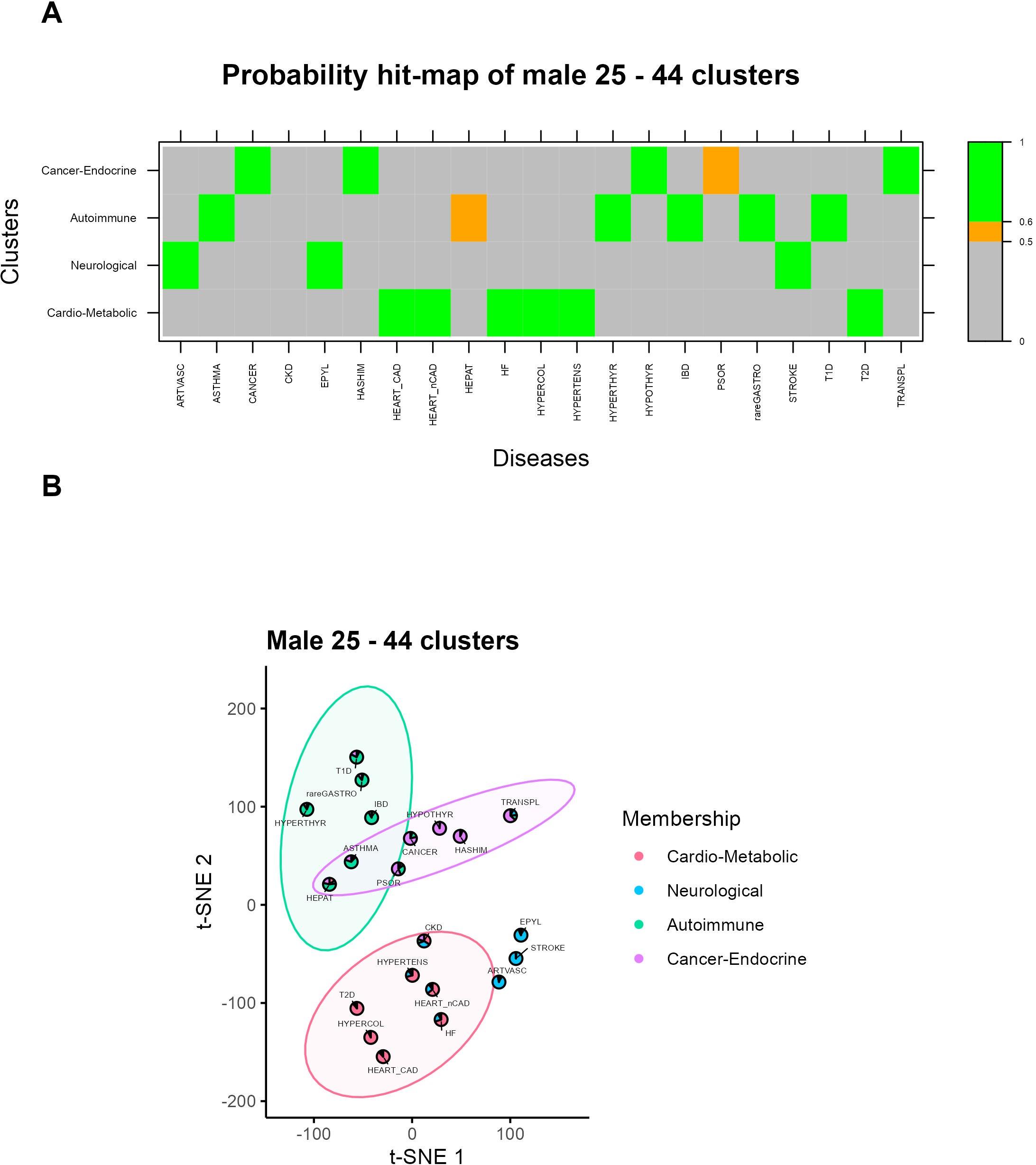


Appendix Figure S6: Clustering analysis, males 45-64. Multimorbidity representation in the population including males aged between 45 and 64 years: A) Hit-map of membership degree of each degree to the clusters. Colors identify thresholds of the membership degrees gray (<0,5), orange (0,5-0,6) and green (>0,6); B) Scatter pie plot of identified clusters in two dimensions using t-SNE and FkM. The pie related to each disease is colored proportionally to the membership degrees of that disease to the clusters.


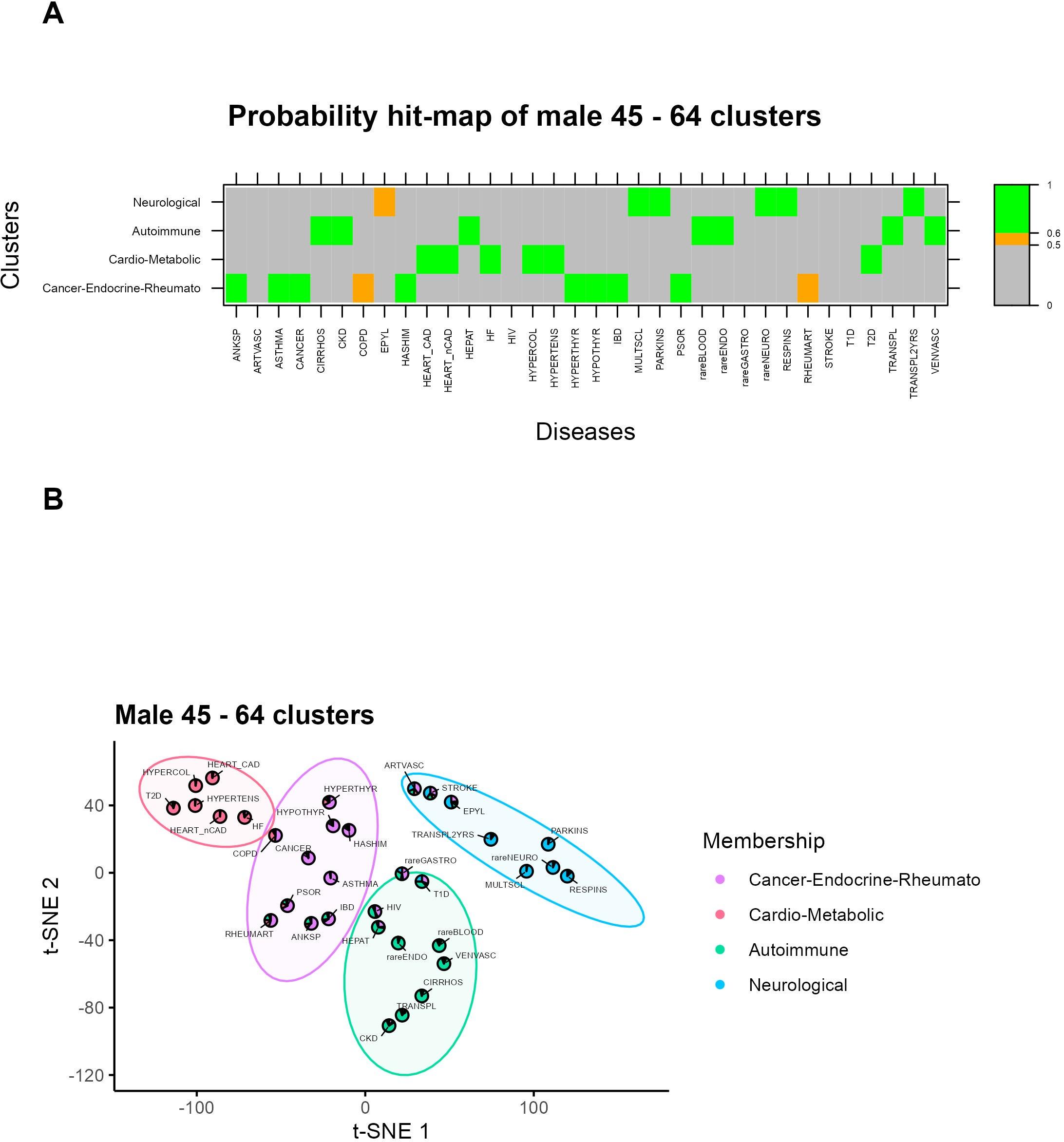


Appendix Figure S7: Clustering analysis, males 65-84. Multimorbidity representation in the population including males aged between 65 and 84 years: A) Hit-map of membership degree of each degree to the clusters. Colors identify thresholds of the membership degrees gray (<0,5), orange (0,5-0,6) and green (>0,6); B) Scatter pie plot of identified clusters in two dimensions using t-SNE and FkM. The pie related to each disease is colored proportionally to the membership degrees of that disease to the clusters.


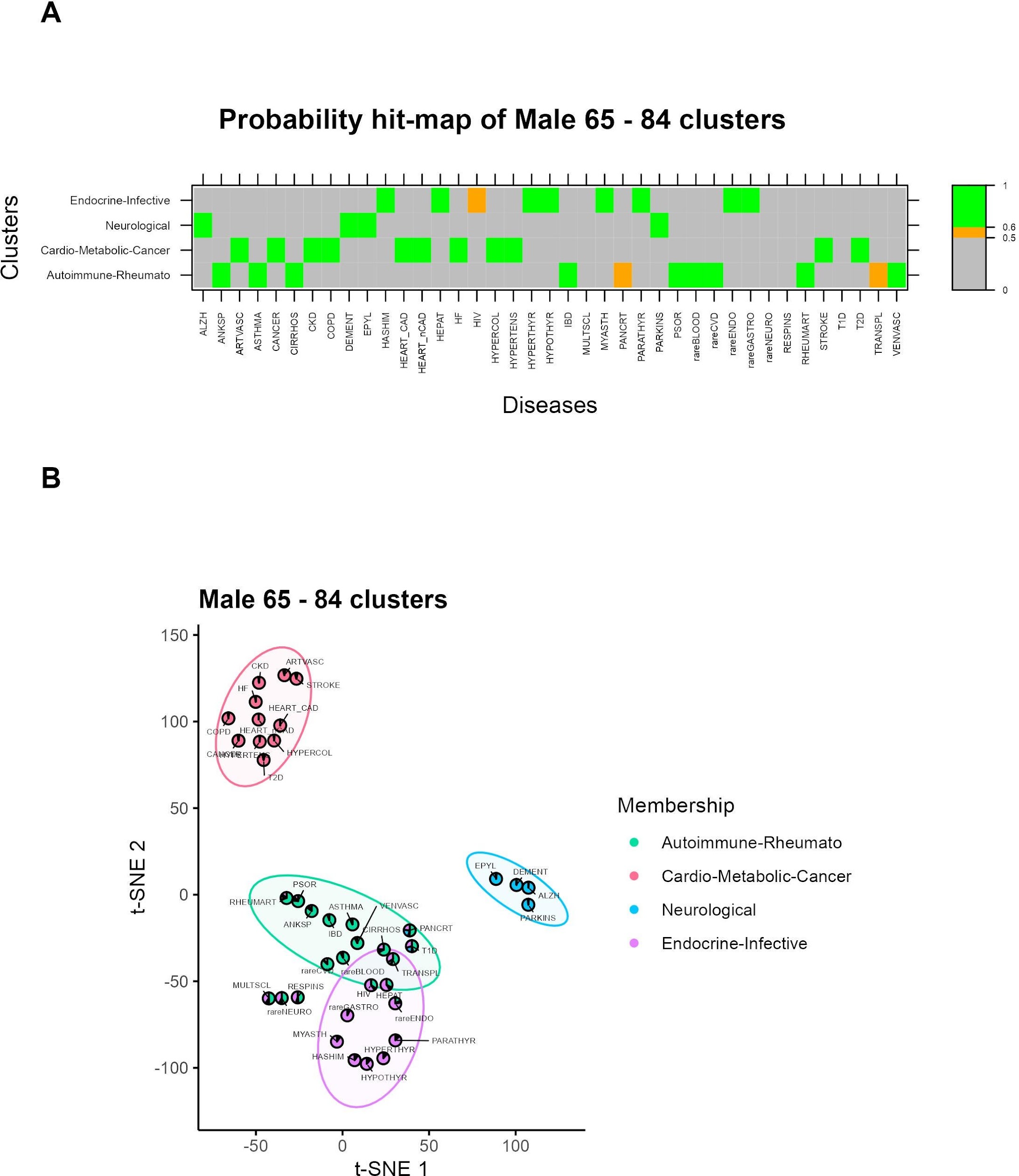


.

Appendix Figure S8: Clustering analysis, males 85-100. Multimorbidity representation in the population including males aged between 85 and 100 years: A) Hit-map of membership degree of each degree to the clusters. Colors identify thresholds of the membership degrees gray (<0,5), orange (0,5-0,6) and green (>0,6); B) Scatter pie plot of identified clusters in two dimensions using t-SNE and FkM. The pie related to each disease is colored proportionally to the membership degrees of that disease to the clusters.


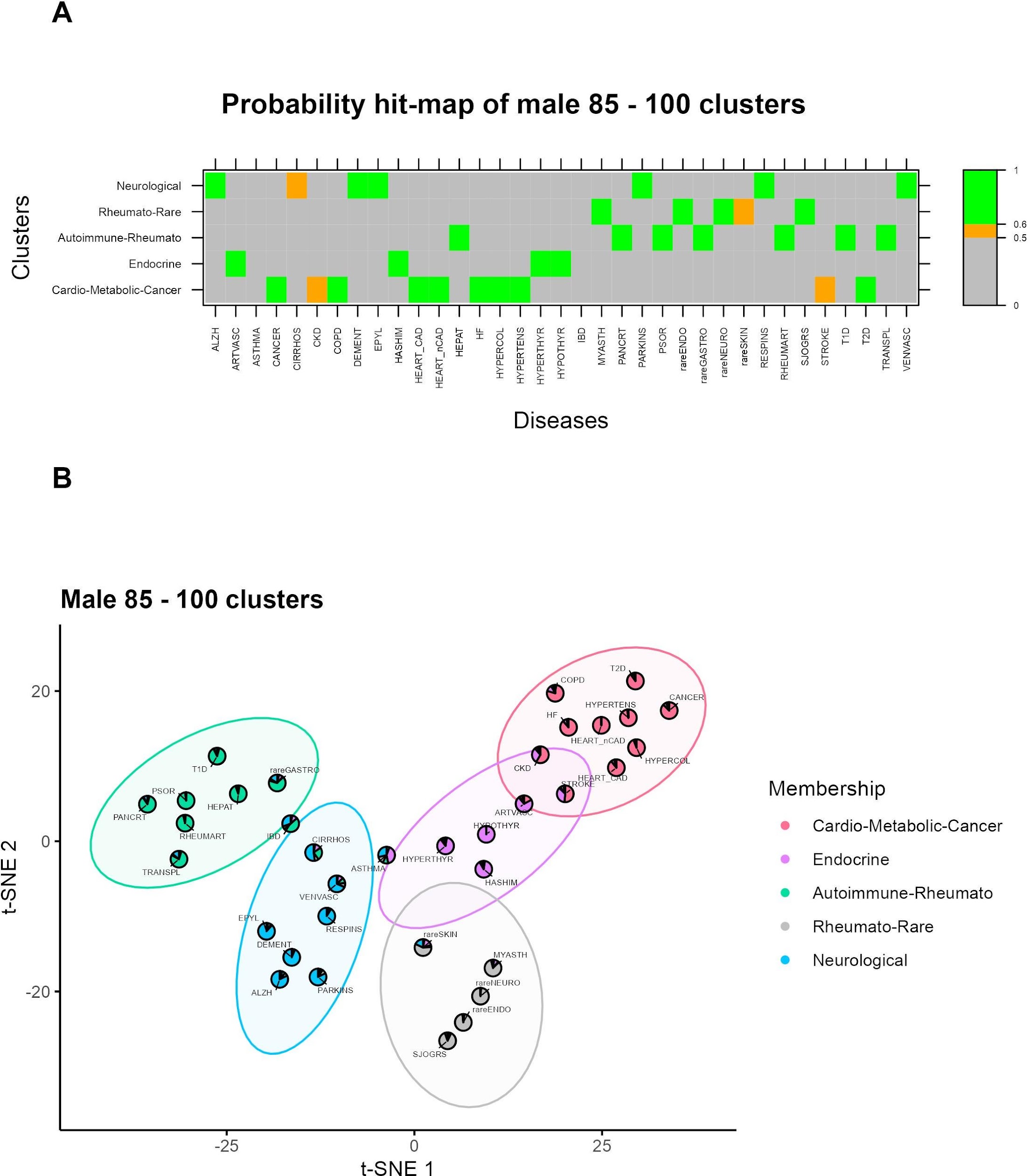


- 1. **Sub-clustering**

Appendix Table S4: Sub-clustering analysis, males and females 25-44. Numbers in brackets refer to the cluster and sub-cluster prevalences (bold) or to disease specific prevalences (italic). Rare diseases excluded from the analysis are indexed at the bottom.

| **Class of Age** |  | **Male** | | | | |  | **Female** | | | | |
| --- | --- | --- | --- | --- | --- | --- | --- | --- | --- | --- | --- | --- |
| **25 - 44** |  | **Cluster attributes** | | | | |  | **Cluster attributes** | | | | |
|  |  | **Cardio-Metabolic** | **Cancer-Endocrine** | **Autoimmune** | **Neurological** | **Not Assigned** |  | **Cancer-Endocrine** | **Cardio-Metabolic** | **Autoimmune** | **Neurological** | **Not Assigned** |
|  |  | **(24.13%)** | **(16.79%)** | **(5.46%)** | **(2.34%)** |  |  | **(22.30%)** | **(6.55%)** | **(2.06%)** | **(2.02%)** |  |
|  |  | **Sub-c1**  **(12.18%)** | **Sub-c1**  **(0.04%)** | **Sub-c1**  **(1.4%)** | ARTVASC  *(0.07%)* | CKD  *(0.12%)* |  | **Sub-c1**  **(17.82%)** | **Sub-c1**  **(0.61%)** | ASTHMA  *(0.41%)* | **Sub-c1**  **(0.14%)** | HASHIM  *(1.15%)* |
|  |  | HEART_nCAD *(0.31%)* | HASHIM  *(0.18%)* | HYPERTHYR *(0.06%)* | EPYL  *(0.09%)* |  |  | CANCER  *(0.73%)* | HYPERCOL *(0.10%)* | rareGASTRO *(0.22%)* | rareENDO  *(0.06%)* | HEPAT  *(0.06%)* |
|  |  | HF  *(0.08%)* | TRANSPL  *(0.06%)* | IBD  *(0.09%)* | STROKE  *(0.09%)* |  |  | HYPERTHYR *(0.25%)* | T2D  *(0.15%)* | T1D  *(0.09%)* | VENVASC  *(0.05%)* | LUPUS  *(0.05%)* |
|  |  | HYPERTENS *(0.51%)* |  | rareGASTRO *(0.06%)* |  |  |  | HYPOTHYR *(1.48%)* |  |  |  | REUMA  *(0.05%)* |
|  |  |  |  | T1D  *(0.08%)* |  |  |  | MULTSCL  *(0.09%)* |  |  |  |  |
|  |  | **Sub-c2**  **(5.57%)** | **Sub-c2**  **(6.91%)** | **Sub-c2**  **(0.83%)** |  |  |  | **Sub-c2**  **(0.49%)** | **Sub-c2**  **(3.79%)** |  | **Sub-c2**  **(1.26%)** |  |
|  |  | HEART_CAD *(0.09%)* | CANCER  *(0.36%)* | ASTHMA  *(0.27%)* |  |  |  | IBD  *(0.10%)* | CKD  *(0.08%)* |  | ARTVASC  *(0.07%)* |  |
|  |  | HYPERCOL *(0.22%)* | HYPOTHYR *(0.31%)* | HEPAT  *(0.08%)* |  |  |  | PSOR  *(0.07%)* | HEART_nCAD *(0.23%)* |  | EPYL  *(0.10%)* |  |
|  |  | T2D  *(0.13%)* | PSOR  *(0.05%)* |  |  |  |  | RHEUMART *(0.08%)* | HYPERTENS *(0.33%)* |  | STROKE  *(0.10%)* |  |
|  |  | Prevalence < 0.05% (ALZH, ANKSP, CIRRHOS, COPD, DEMENT, HIV, LUPUS, MULTSCL, MYASTH, PANCRT, PARATHYR, PARKINS, rareBLOOD, rareCONGEN, rareCVD, rareENDO, rareNEURO, rareSKIN, RESPINS, REUMA, RHEUMART, SJOGRS, SYSTSCL, TRANSPL2YRS, VENVASC). | | | | |  | Prevalence < 0.05% (ALZH, ANKSP, CIRRHOS, COPD, DEMENT, HEART_CAD ,HF, HIV, MYASTH, PANCRT, PARATHYR, PARKINS, rareBLOOD, rareCONGEN, rareCVD, rareNEURO, rareSKIN, RESPINS, SJOGRS, SYSTSCL, TRANSPL2YRS, TRANSPL). | | | | |
|  |  |  |  |  |  |  |  |  |  |  |  |  |

Appendix Table S5: Sub-clustering analysis, males and females 45-64. Numbers in brackets refer to the cluster and sub-cluster prevalences (bold) or to disease specific prevalences (italic). Rare diseases excluded from the analysis are indexed at the bottom.

| **Class of Age** |  | **Male** | | | | |  | **Female** | | | | |
| --- | --- | --- | --- | --- | --- | --- | --- | --- | --- | --- | --- | --- |
| **45 - 64** |  | **Cluster attributes** | | | | |  | **Cluster attributes** | | | | |
|  |  | **Cardio-Metabolic** | **Cancer-Endocrine-Rheumato** | **Autoimmune** | **Neurological** | **Not Assigned** |  | **Cardio-Metabolic-Cancer-Endocrine** | **Neurological** | **Rheumato** | **Autoimmune-Infective** | **Not Assigned** |
|  |  | **(60.10%)** | **(10.41%)** | **(1.97%)** | **(0.23%)** |  |  | **(80.85%)** | **(2.01%)** | **(1.64%)** | **(0.77%)** |  |
|  |  | **Sub-c1**  **(4.72%)** | **Sub-c1**  **(6.63%)** | **Sub-c1**  **(1.07%)** | **Sub-c1**  **(0.01%)** | ARTVASC  *(0.67%)* |  | **Sub-c1**  **(3.52%)** | **Sub-c1**  **(1.68%)** | **Sub-c1**  **(0.34%)** | **Sub-c1**  **(0.51%)** | PARATHYR *(0.11%)* |
|  |  | HEART_nCAD *(2.80%)* | CANCER  *(2.17%)* | CIRRHOS  *(0.33%)* | EPYL  *(0.25%)* | HIV  *(0.25%)* |  | COPD  *(0.63%)* | ARTVASC  *(0.35%)* | ANKSP  *(0.10%)* | CIRRHOS  *(0.20%)* | rareBLOOD *(0.09%)* |
|  |  | HF  *(0.95%)* | COPD  *(0.73%)* | CKD  *(0.56%)* | TRANSPL2YRS *(0.06%)* | rareGASTRO *(0.10%)* |  | HEART_CAD *(0.55%)* | EPYL  *(0.23%)* | IBD  *(0.27%)* | CKD  *(0.31%)* | rareGASTRO *(0.28%)* |
|  |  |  | HASHIM  *(0.39%)* | TRANSPL  *(0.20%)* |  | STROKE  *(0.81%)* |  | HEART_nCAD *(1.44%)* | STROKE  *(0.59%)* | PSOR  *(0.30%)* | T1D  *(0.08%)* |  |
|  |  |  | HYPERTHYR *(0.23%)* |  |  | T1D  *(0.09%)* |  | HF  *(0.37%)* | VENVASC  *(0.20%)* |  | TRANSPL  *(0.10%)* |  |
|  |  |  | HYPOTHYR *(1.00%)* |  |  |  |  |  |  |  |  |  |
|  |  |  |  |  |  |  |  |  |  |  |  |  |
|  |  | **Sub-c2**  **(47.89%)** | **Sub-c2**  **(0.76%)** | **Sub-c2**  **(0.42%)** | **Sub-c2**  **(0.11%)** |  |  | **Sub-c2**  **(35.74%)** | **Sub-c2**  **(0.06%)** | **Sub-c2**  **(0.86%)** | **Sub-c2**  **(0.13%)** |  |
|  |  | HEART_CAD *(2.28%)* | ANKSP  *(0.08%)* | HEPAT  *(0.61%)* | MULTSCL  *(0.12%)* |  |  | ASTHMA  *(1.04%)* | MULTSCL  *(0.24%)* | LUPUS  *(0.12%)* | HEPAT  *(0.35%)* |  |
|  |  | HYPERCOL *(4.37%)* | ASTHMA  *(0.58%)* | rareBLOOD *(0.06%)* | PARKINS  *(0.09%)* |  |  | CANCER  *(3.51%)* | PARKINS  *(0.06%)* | rareCONGEN *(0.06%)* | HIV  *(0.09%)* |  |
|  |  | HYPERTENS *(8.31%)* | IBD  *(0.34%)* | rareENDO  *(0.10%)* | rareNEURO *(0.08%)* |  |  | HYPERCOL *(2.29%)* | rareNEURO *(0.09%)* | rareENDO  *(0.13%)* |  |  |
|  |  | T2D  *(2.96%)* | PSOR  *(0.28%)* | VENVASC  *(0.22%)* | RESPINS  *(0.07%)* |  |  | HYPERTENS *(6.30%)* |  | REUMA  *(0.14%)* |  |  |
|  |  |  | RHEUMART *(0.12%)* |  |  |  |  | T2D  *(1.97%)* |  | RHEUMART *(0.32%)* |  |  |
|  |  |  |  |  |  |  |  | **Sub-c3 (19.37%)** |  | SJOGRS  *(0.18%)* |  |  |
|  |  |  |  |  |  |  |  | HASHIM  *(2.81%)* |  | SYSTSCL  *(0.07%)* |  |  |
|  |  |  |  |  |  |  |  | HYPERTHYR *(0.88%)* |  |  |  |  |
|  |  |  |  |  |  |  |  | HYPOTHYR *(4.68%)* |  |  |  |  |
|  |  | Prevalence < 0.05% (ALZH, DEMENT, LUPUS, MYASTH, PANCRT, PARATHYR, rareCONGEN , rareCVD, rareSKIN, REUMA, SJOGRS, SYSTSCL). | | | | |  | Prevalence < 0.05% (ALZH, DEMENT, MYASTH, PANCRT, rareCVD, rareSKIN, RESPINS, TRANSPL2YRS). | | | | |
|  |  |  |  |  |  |  |  |  |  |  |  |  |

Appendix Table S6: Sub-clustering analysis, males and females 65-84. Numbers in brackets refer to the cluster and sub-cluster prevalences (bold) or to disease specific prevalences (italic). Rare diseases excluded from the analysis are indexed at the bottom.

| **Class of Age** |  | **Male** | | | | |  | **Female** | | | | | |
| --- | --- | --- | --- | --- | --- | --- | --- | --- | --- | --- | --- | --- | --- |
| **65 - 84** |  | **Cluster attributes** | | | | |  | **Cluster attributes** | | | | | |
|  |  | **Cardio-Metabolic-Cancer** | **Endocrine-Infective** | **Autoimmune-Rheumato** | **Neurological** | **Not assigned** |  | **Cardio-Metabolic-Cancer-Endocrine** | **Neurological** | **Rheumato** | **Bowel-Autoimmune** | **Pulmonary-Autoimmune** | **Not assigned** |
|  |  | **(91.74%)** | **(1.35%)** | **(0.66%)** | **(0.62%)** |  |  | **(89.27%)** | **(3.97%)** | **(0.72%)** | **(0.03%)** | **(0.03%)** |  |
|  |  | **Sub-c1**  **(7.31%)** | **Sub-c1**  **(0.07%)** | **Sub-c1**  **(0.15%)** | ALZH  *(0.67%)* | MULTSCL  *(0.11%)* |  | **Sub-c1 (59.59%)** | **Sub-c1 (2.21%)** | **Sub-c1 (0.05%)** | ANKSP  *(0.08%)* | **Sub-c1 (0.002%)** | ASTHMA *(1.71%)* |
|  |  | ARTVASC  *(4.28%)* | HEPAT  *(0.87%)* | ANKSP  *(0.11%)* | DEMENT  *(0.43%)* | rareNEURO *(0.15%)* |  | CANCER *(11.08%)* | ARTVASC *(1.54%)* | CIRRHOS *(0.42%)* | IBD  *(0.45%)* | PANCRT *(0.08%)* | HEPAT  *(0.78%)* |
|  |  | CKD  *(3.35%)* | HIV  *(0.19%)* | IBD  *(0.85%)* | EPYL  *(0.65%)* | RESPINS  *(0.31%)* |  | HYPERCOL *(15.39%)* | CKD  *(1.42%)* | rareCVD  *(0.09%)* | rareSKIN *(0.05%)* | T1D  *(0.09%)* | PARATHYR *(0.22%)* |
|  |  | HF  *(5.96%)* | rareENDO  *(0.16%)* | PSOR  *(0.55%)* | PARKINS  *(1.22%)* | T1D  *(0.14%)* |  | HYPERTENS *(33.61%)* | STROKE *(3.27%)* | rareENDO *(0.14%)* |  |  | rareBLOOD *(0.07%)* |
|  |  | STROKE  *(5.38%)* | rareGASTRO *(0.08%)* | RHEUMART *(0.39%)* |  |  |  | T2D  *(10.81%)* | VENVASC *(0.57%)* | TRANSPL *(0.12%)* |  |  | rareGASTRO *(0.17%)* |
|  |  |  |  |  |  |  |  |  |  |  |  |  |  |
|  |  | **Sub-c2**  **(87.29%)** | **Sub-c2**  **(1.13%)** | **Sub-c2**  **(0.30%)** |  |  |  | **Sub-c2 (10.20%)** | **Sub-c2 (0.69%)** | **Sub-c2 (0.55%)** |  | **Sub-c2 (0.02%)** |  |
|  |  | CANCER  *(13.83%)* | HASHIM  *(0.51%)* | ASTHMA  *(1.06%)* |  |  |  | COPD  *(3.93%)* | ALZH  *(0.86%)* | LUPUS  *(0.12%)* |  | MYASTH *(0.05%)* |  |
|  |  | COPD  *(6.28%)* | HYPERTHYR *(0.64%)* | CIRRHOS  *(0.60%)* |  |  |  | HEART_CAD *(3.79%)* | DEMENT *(0.45%)* | MULTSCL *(0.18%)* |  | rareNEURO *(0.13%)* |  |
|  |  | HEART_CAD *(11.71%)* | HYPOTHYR *(2.75%)* | PANCRT  *(0.12%)* |  |  |  | HEART_nCAD *(9.13%)* | EPYL  *(0.57%)* | RHEUMART *(0.91%)* |  | RESPINS *(0.26%)* |  |
|  |  | HEART_nCAD *(15.16%)* | MYASTH  *(0.08%)* | rareBLOOD *(0.08%)* |  |  |  | HF  *(3.57%)* | PARKINS *(0.83%)* | SYSTSCL *(0.17%)* |  |  |  |
|  |  | HYPERCOL *(21.34%)* | PARATHYR *(0.06%)* | rareCVD  *(0.06%)* |  |  |  |  |  | SJOGRS  *(0.51%)* |  |  |  |
|  |  | HYPERTENS *(40.16%)* |  | TRANSPL  *(0.26%)* |  |  |  | **Sub-c3 (6.28%)** |  | PSOR  *(0.55%)* |  |  |  |
|  |  | T2D  *(15.20%)* |  | VENVASC  *(0.78%)* |  |  |  | HASHIM *(3.02%)* |  | REUMA  *(0.16%)* |  |  |  |
|  |  |  |  |  |  |  |  | HYPERTHYR *(1.52%)* |  |  |  |  |  |
|  |  |  |  |  |  |  |  | HYPOTHYR *(10.10%)* |  |  |  |  |  |
|  |  | Prevalence < 0.05% (LUPUS, rareCONGEN, rareSKIN, REUMA, SJOGRS, SYSTSCL, TRANSPL2YRS). | | | | |  | Prevalence < 0.05% (HIV, rareCONGEN, TRANSPL2YRS). | | | | | |
|  |  |  |  |  |  |  |  |  |  |  |  |  |  |

Appendix Table S7: Sub-clustering analysis, males and females 85-100. Numbers in brackets refer to the cluster and sub-cluster prevalences (bold) or to disease specific prevalences (italic). Rare diseases excluded from the analysis are indexed at the bottom.

| **Class of Age** |  | **Male** | | | | | |  | **Female** | | | | | |
| --- | --- | --- | --- | --- | --- | --- | --- | --- | --- | --- | --- | --- | --- | --- |
| **85 – 100** |  | **Cluster attributes** | | | | | |  | **Cluster attributes** | | | | | |
|  |  | **Cardio-Metabolic-Cancer** | **Neurological** | **Endocrine** | **Autoimmune- Rheumato** | **Rheumato-Rare** | **Not assigned** |  | **Cardio-Metabolic-Cancer** | **Neurological** | **Autoimmune- Rheumato** | **Endocrine** | **Rare** | **Not assigned** |
|  |  | **(94.16%)** | **(2.21%)** | **(0.66%)** | **(0.06%)** | **(0.01%)** |  |  | **(89.00%)** | **(6.63%)** | **(0.24%)** | **(0.04%)** | **(0.001%)** |  |
|  |  | **Sub-c1 (16.87%)** | **Sub-c1 (0.09%)** | ARTVASC *(8.20%)* | **Sub-c1 (0.01%)** | MYASTH *(0.14%)* | ASTHMA *(0.81%)* |  | **Sub-c1 (63.01%)** | **Sub-c1 (3.75%)** | **Sub-c1 (0.10%)** | **Sub-c1 (0.01%)** | MYASTH *(0.06%)* | ARTVASC *(3.25%)* |
|  |  | CKD  *(12.18%)* | CIRRHOS *(0.58%)* | HASHIM *(0.18%)* | HEPAT  *(0.75%)* | rareENDO *(0.09%)* | IBD  *(0.89%)* |  | CANCER *(15.62%)* | DEMENT *(4.82%)* | CIRRHOS *(0.43%)* | HYPERTHYR *(0.50%)* | rareNEURO *(0.05%)* | CKD  *(6.08%)* |
|  |  | COPD  *(18.46%)* | RESPINS *(1.25%)* | HYPERTHYR *(0.42%)* | rareGASTRO *(0.05%)* | rareNEURO *(0.10%)* |  |  | HYPERCOL *(23.04%)* | EPYL  *(1.21%)* | PSOR  *(0.18%)* | PARATHYR *(0.08%)* |  | HASHIM *(0.48%)* |
|  |  | HF  *(21.27%)* | VENVASC *(1.60%)* | HYPOTHYR *(4.58%)* | T1D  *(0.25%)* | rareSKIN *(0.06%)* |  |  | HYPERTENS *(66.60%)* | STROKE *(12.62%)* | RHEUMART *(0.71%)* |  |  | PANCRT *(0.15%)* |
|  |  | STROKE *(17.30%)* |  |  |  | SJOGRS *(0.08%)* |  |  | HYPOTHYR *(10.95%)* |  | SJOGRS *(0.37%)* |  |  |  |
|  |  |  |  |  |  |  |  |  | T2D  *(18.08%)* |  | SYSTSCL *(0.11%)* |  |  |  |
|  |  | **Sub-c2 (83.42%)** | **Sub-c2 (1.68%)** |  | **Sub-c2 (0.03%)** |  |  |  | **Sub-c2 (23.55%)** | **Sub-c2 (0.55%)** | **Sub-c2**  **(0.05%)** | **Sub-c2 (0.01%)** |  |  |
|  |  | CANCER *(30.05%)* | ALZH  *(3.75%)* |  | PANCRT *(0.19%)* |  |  |  | COPD  *(9.18%)* | ALZH  *(4.51%)* | ASTHMA *(0.94%)* | rareCVD *(0.07%)* |  |  |
|  |  | HEART_CAD *(23.42%)* | DEMENT *(3.85%)* |  | PSOR  *(0.30%)* |  |  |  | HEART_CAD *(11.94%)* | PARKINS *(2.76%)* | HEPAT  *(0.66%)* | VENVASC *(1.44%)* |  |  |
|  |  | HEART_nCAD *(41.29%)* | EPYL  *(1.47%)* |  | RHEUMART *(0.48%)* |  |  |  | HEART_nCAD  *(28.02%)* | RESPINS *(1.28%)* | IBD  *(0.44%)* |  |  |  |
|  |  | HYPERCOL *(34.47%)* | PARKINS *(4.31%)* |  | TRANSPL *(0.05%)* |  |  |  | HF  *(17.09%)* |  | rareGASTRO *(0.08%)* |  |  |  |
|  |  | HYPERTENS *(81.47%)* |  |  |  |  |  |  |  |  | T1D  *(0.22%)* |  |  |  |
|  |  | T2D  *(24.64%)* |  |  |  |  |  |  |  |  |  |  |  |  |
|  |  |  |  |  |  |  |  |  |  |  |  |  |  |  |
|  |  | Prevalence < 0.05% (ANKSP, HIV, LUPUS, MULTSCL, PARATHYR, rareBLOOD, rareCONGEN, rareCVD, REUMA, SYSTSCL, TRANSPL2YRS). | | | | | |  | Prevalence < 0.05% (ANKSP, HIV, LUPUS, MULTSCL, rareBLOOD, rareCONGEN, rareENDO, rareSKIN, REUMA, TRANSPL, TRANSPL2YRS). | | | | | |
